# Multiple modes of transcriptional regulation by the nuclear hormone receptor RARγ in human squamous cell carcinoma

**DOI:** 10.1016/j.jbc.2025.110965

**Published:** 2025-11-20

**Authors:** Helen R. Hoxie, Xiao-Han Tang, Lorraine J. Gudas

**Affiliations:** 1 Department of Pharmacology, Weill Cornell Medicine, New York, New York, USA; 2 Sandra and Edward Meyer Cancer Center, Weill Cornell Medicine, New York, New York, USA; 3 Weill Cornell Graduate School of Medical Sciences, Cornell University, New York, New York, USA

**Keywords:** nuclear receptors, retinoic acid, head and neck squamous cell carcinoma (HNSCC), epithelial cell, transcription regulation

## Abstract

Vitamin A metabolism and signaling through nuclear retinoic acid receptors (RARs α,β,γ) regulate embryogenesis, immune functions, and cell differentiation in most cell types. RARγ is highly expressed in stratified squamous epithelial cells of the oral cavity and skin. Although data indicate that RARγ agonism is antitumorigenic in oral cavity squamous cell carcinoma (OCSCC), the specific, primary gene targets of RARγ remain poorly characterized. Here, we define RARγ signaling pathways through integrating genome-wide RARγ binding by Cleavage under Targets and Release Using Nuclease (CUT&RUN), chromatin histone marks, and global transcriptomics ± agonists in human OCSCC cells and in human OCSCC cells with deletion of RARG (gene for RARγ) (RARGKO). Notably, transcripts for some genes associated with stratified squamous cell differentiation, including NOTCH1, NOTCH3, and the NOTCH ligands, JAG2 and DLL1, were reduced in RARGKO without added ligand. Loss of RARγ binding also reduced expression of a broad group of genes that regulates cell identity and extracellular matrix communication, as well as the retinaldehyde reductase, DHRS3, a crucial retinol homeostasis regulating enzyme. We also discovered targets that were directly repressed by RARγ and thus showed higher expression in RARGKO cells. We identified RARG, PPARG, and RXRA as direct RARγ gene targets, indicating that RARγ could control transcription of other genes *via* regulation of RXRα, a transcription factor with multiple dimerization partners, in OCSCC. Taken together, RARγ-mediated transcriptional regulation is multifaceted and context-dependent. The delineation of these key RARγ targets and signaling pathways should allow the development of new therapeutics for OCSCC.

Vitamin A (all-trans retinol, VA) is a critical micronutrient obtained by mammals through their diet (1). The levels of VAs’ metabolites, particularly the bioactive metabolites *all-trans* retinoic acid (RA) and 4-oxo-RA, are tightly regulated in various cell types (2). RA is an endogenous agonist for the nuclear retinoic acid receptors alpha, beta, and gamma (RARα, RARβ, and RARγ), which are generally characterized as ligand-activated receptors that contain both a DNA-binding domain and a ligand-binding domain that dimerizes with retinoid X receptors (RXRs) on their target DNA regions, retinoic acid response element (RAREs) (3, 4, 5) (Fig. 1, *A* and *B*). Binding of RA to RARs induces an exchange of the regulatory proteins constitutively bound to RARs with regulators that activate transcriptional programs involved in a wide variety of cellular processes (6). RA signaling through RARs is particularly important in regulating processes such as cell growth, differentiation, immunity, hematopoiesis, and ocular functions (7, 8, 9, 10, 11).

**Figure 1:**
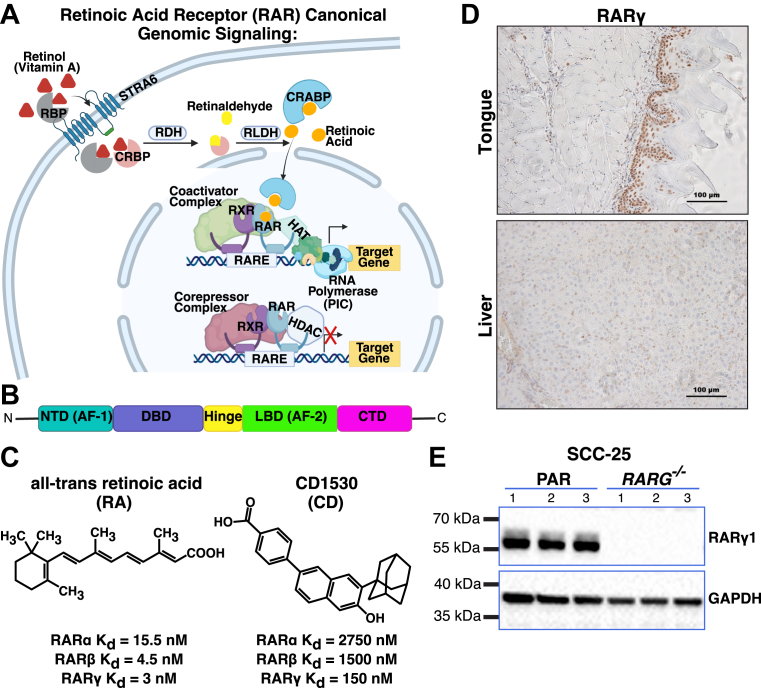
**Approach to identify RARγ gene targets in the context of squamous cell carcinoma**. *A*, canonical model for retinoic acid receptor signaling. *B*, general structure of RARs showing the N -terminal and ligand binding domains (LBD), as well as a zinc-finger DNA binding domain (DBD). *C*, chemical structures and dissociation constants for endogenous pan-RAR ligand (RA) and synthetic RARγ-selective agonist (CD1530). *D*, identification of RARγ tissue localization with staining in male C57BL/6 mouse tongue *(Top)* and liver *(Bottom)*. The scale bars represent 100 μm distances. *E*, confirmation of protein-level deletion of RARγ in SCC-25 RARGKO cells by western blotting of PAR (parental) and RARGKO cells seeded in triplicate and collected the following day. RAR, retinoic acid receptor.

Mouse RAR knockout models demonstrated severe abnormalities in double null mutant mice offspring (lacking two RARα/β/γ isotypes), many of which mimicked a VA deficiency state (12, 13, 14). Much of the work on RA signaling has been done in embryonic stem cells, through which both functional redundancies between the RARs as well as isotype-specific developmental requirements have been identified (12, 15).

The broad impact of RA signaling on growth and differentiation has led to targeting RAR actions for the prevention and/or treatment of several different cancers (16), but RA bioavailability and RAR isotype expression levels and functionality vary greatly among different cell types. RARα and RARγ are expressed in most cell types in the body, including the squamous epithelial cells of the oral cavity and skin (17, 18). RA binds as an agonist with high affinity (K_d_ < 15 nM) to all RAR isotypes (Fig. 1*C*). In therapeutic targeting of RAR-mediated functions, several synthetic modulators of RAR signaling have been developed to selectively target RAR isotypes, such as the RARγ-selective agonist CD1530 (19) (Fig. 1*C*).

Oral cavity squamous cell carcinoma (OCSCC), which originates from oral epithelia, is a common type of head and neck cancer (20, 21), and rates of OCSCC are increasing (22). Risk factors such as tobacco use, alcohol, immunodeficiency, poor oral health, and genetic predispositions can cause the oral mucosal layer to accumulate key transforming mutations, including mutations that inactivate the Notch tumor suppressor gene (23), leading to tumor development and ultimately invasion through the basement membrane. RARγ suppresses transformation/tumorigenesis in mouse keratinocytes, and RA-induced apoptosis and cell-cycle arrest in transformed mouse keratinocytes depend on a functional RARγ; moreover, RARγ expression decreases during human squamous cell carcinoma (SCC) progression (24, 25). Further studies in a mouse model of OCSCC in our laboratory showed that combinatorial pharmacological treatment of RARγ with the agonist CD1530 and RXR with the agonist bexarotene attenuated carcinogenesis in mice treated with 4-nitroquinoline 1-oxide, a water-soluble carcinogen (26). Interestingly, while pharmacological antagonism of RARγ can drive necroptosis in cancer stem cells, mouse skin keratinocytes lacking RARγ are resistant to DNA damage-induced necroptosis (27, 28). In summary, the data to date indicate a tumor suppressive function for RARγ in OCSCC.

We aimed to elucidate the genomic and transcriptomic consequences of RARγ activity in OCSCC. Since CD1530 has been shown to reduce the development of oral cavity SCC, here, we explored the gene expression pathways regulated by RARγ in a human oral cavity SCC line to elucidate how RARγ achieves these positive therapeutic results. Using a CRISPR-Cas9-mediated KO of RARγ and pharmacological treatments with RAR agonists, we identified RARγ-specific gene targets and pathways, as well as targets that overlap with other RA-responsive nuclear receptors. In addition, we delineated how RARγ influences chromatin accessibility and modification of histones, providing insights into RARγ's role in modulating the transcriptome in OCSCC cells.

## Results

### RNA-seq in parental and RARGKO SCC-25 cells without added agonists reveals key pathways regulated by RARγ

**Figure 2:**
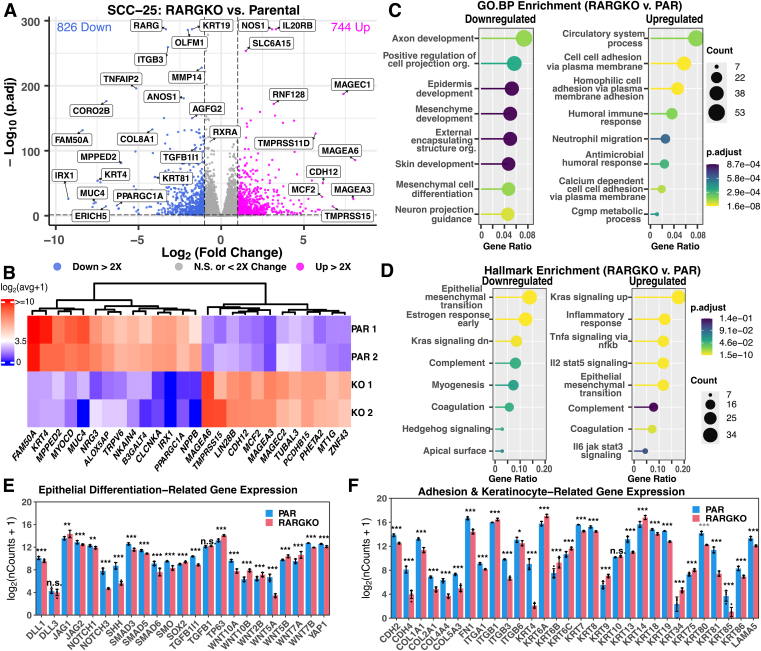
**RNA-seq reveals transcriptome-wide changes in SCC-25 cells lacking RARγ**. *A*, differential expression of genes upregulated *(pink)* and downregulated *(blue)* in RARGKO with respect to parental (p.adj < 0.05, |log2FC| > 1). *B*, heatmap of log-transformed mean expression values for genes that are conditionally silenced (mean DESeq2-normalized counts < 10) or activated (>50) in cells that lack or express RARγ. *C*, ClusterProfiler results of Gene Ontology enrichment analyses on the set of genes upregulated (*left*) or downregulated > 2-fold (*right*) in RARGKO *versus* parental cells (p.adj < 0.05). *Top* pathways by gene ratio (genes in the pathway/genes in the set) are shown (genes from input list matching the GO term); *D*, hallmark pathway enrichment of the set of genes upregulated (*left*) or downregulated > 2-fold (*right*) in RARGKO *versus* parental cells. Top 5 pathways by gene ratio are shown. *E* and *F*, gene expression shown as log_2_(DESeq2 size-factor–normalized counts + 1) in SCC-25 cells (bars = mean ± SD, points = individual values, n = 6 biological replicates per group). *Asterisks* indicate DESeq2 adjusted *p* values (Wald test, BH correction; ∗ < 0.05, ∗∗ < 0.01, ∗∗∗ < 0.001, n.s. = not significant). *E*, notch/TGFβ/Wnt-related factors. *F*, substrate-adhesion and epithelial-keratinization genes. BH, Benjamini–Hochberg; GO, Gene Ontology; RAR, retinoic acid receptor; TGF, transforming growth factor.

Immunohistochemistry in mouse tongue samples confirmed robust RARγ protein expression in the oral cavity compared to the liver, with the strongest enrichment in the oral squamous epithelium (Fig. 1*D*). To determine general pathways and gene targets of RARγ signaling in human OCSCC that initiated in the oral epithelium, we cotransfected SCC-25 cells, a human OCSCC cell line, with spCas9 and a guide RNA targeting all RARγ variants (exon 5 in human RARγ1). We subcloned stable knockout (RARGKO) lines and confirmed KO at both the DNA and protein levels (Fig. 1*E*, Supporting Information Fig. S1*A*). We identified changes to the transcriptome with RNA-seq and subsequent differential expression analysis. Principal component analysis confirmed transcriptome wide differences between parental (PAR) and RARGKO (Supporting Information, Fig. S1*B*). Interrogation of other RAR/RXR isotypes confirmed that RARγ is the predominantly expressed RAR in SCC-25 cells, and that RARGKO cells do not compensate for the loss of RARG transcription with proportional upregulation of RARα/β (Supporting Information Table S1). Deletion of RARγ resulted in broad transcript shifts compared to SCC-25 PAR cells—744 transcripts were upregulated more than two-fold in RARGKO compared to PAR (p-adjusted < 0.05) and 826 transcripts were downregulated more than two-fold (p.adj < 0.05) (Fig. 2*A*, Supporting Data S1). We determined that several genes, such as SPRR1B, MAGEA3, and MAGEC2, were virtually silenced (length-normalized counts < 10) in RARGKO cells. In contrast, genes such as KRT4, IRX1, and MUC4 were virtually silenced in parental cells (Fig. 2, *A* and *B*).

Gene Ontology overrepresentation analysis (ORA) (29, 30, 31) was performed on the set of genes differentially regulated more than 2-fold in the absence of RARγ (Fig. 2, *C* and *D*). Both downregulated in RARGKO *versus* PAR and upregulated in RARGKO *versus* PAR gene sets were enriched for factors associated with cellular adhesion and inflammatory responses. The upregulated gene set included genes related to stress pathways that included tumor necrosis factor α (TNFα) signaling, glycolysis, lipid metabolism, and inflammatory response (Fig. 2, *C* and *D*). Notably, the set downregulated in the RARGKO included genes related to differentiation on the transforming growth factor B1 (TGFB1) and Hedgehog axes, such as SMAD3, SMAD6, and SHH (Fig. 2*E*).

Because RARγ is highly expressed in the basal layer squamous epithelial stem cells and RA signaling is involved in differentiation, we focused on differentiation as it relates to the oral stratified squamous epithelium. The Notch receptors, along with their ligands Delta-like and Jagged, are important differentiation mediators in the basal layer of the oral squamous epithelium (32). Squamous epithelium stratification is generally characterized through shifts in differentiation-related transcription factor activity, adhesion molecules, and cytokeratins (33). Notch signaling pathway receptor and ligand mRNAs, including NOTCH1, NOTCH3, JAG2, and DLL1, were downregulated in RARGKO compared to PAR. Notably, however, the transcript for one Notch ligand, Jag1, was upregulated in RARGKO *versus* PAR (Fig. 2*E*). Among abundantly expressed cytokeratins in SCC-25 cells, we observed increases in KRT6A and KRT14 mRNAs, and decreases in KRT7 and KRT80 in RARGKO compared to PAR; we also observed increases in integrin ITGB1 and ITGA6 transcripts, but reductions in ITGB3 and ITGB6 transcripts in RARGKO (Fig. 2*F*).

### Selective RARγ agonist and pan-RAR agonist treatment of SCC-25 PAR cells

**Figure 3:**
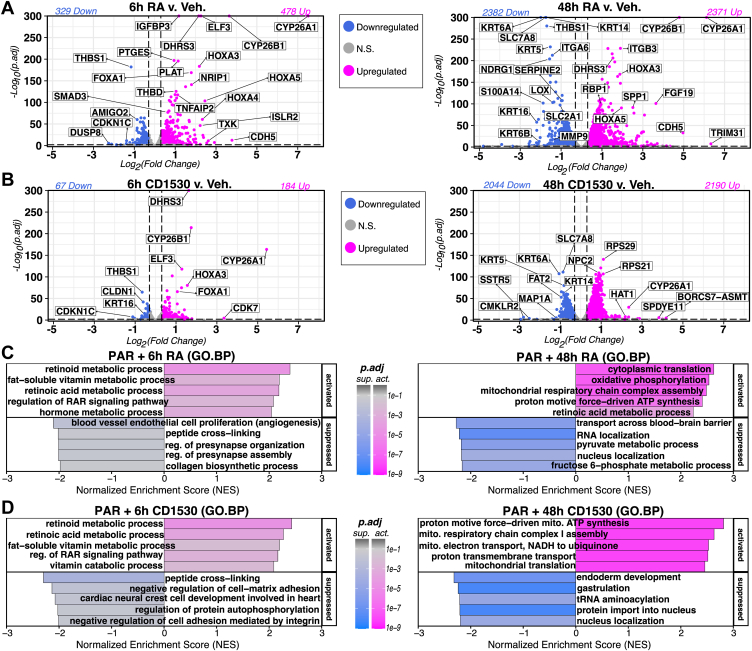
**RNA-Seq of SCC-25 cells (PAR shown) treated in triplicate for 6 h or 48 h with 1 μM RA, 1 μM CD1530, or vehicle control (0.1% DMSO)**. *A*, volcano plots of genes upregulated *(pink)* and downregulated *(blue)* in ATRA-treated SCC-25 cells at 6 h (*left*) and 48 h *(right*). *B*, volcano plots of genes upregulated *(pink)* and downregulated *(blue)* in CD1530-treated SCC-25 cells at 6 h (*left*) and 48 h *(right*); (cutoffs set to p.adj < 0.01, |log_2_FC| > 0.3). *C* and *D*, bar plots illustrating GSEA results on stat-sorted DESeq2 results with the MSigdb Gene Ontology: Biological Process (GO.BP) annotation database for RA treatments in PAR at 6 h (*left*) and 48 h (*right*) in response to (*C*) 1 μM RA treatment or (*D*) 1 μM CD1530 treatment. ATRA, *All-trans* retinoic acid; DMSO, dimethyl sulfoxide; GSEA, gene set enrichment analysis; RA, retinoic acid..

We further examined the changes in transcript levels in SCC-25 PAR cells in the presence of two different agonists. *All-trans* retinoic acid (ATRA/RA) is the endogenous pan-RAR agonist that binds all three RARs, while CD1530 displays selectivity for RARγ (19) (Fig. 1*C*). SCC-25 PAR cells were treated with either 1 μM RA, 1 μM CD1530, or 0.1% dimethyl sulfoxide (DMSO) vehicle, and after 6 h (early stage) and 48 h (late stage), total RNA was extracted, and the rRNA-depleted complementary DNA (cDNA) libraries for each sample were sequenced (Fig. 3 and Supporting Information Data S1). To capture smaller but meaningful changes after agonist treatments, we employed a |log_2_(FC)| cutoff of 0.3 with a more stringent p.adj cutoff of 0.01 to define significantly changed transcripts.

After RA treatment of PAR cells, 478 transcripts at 6 h and 2371 transcripts at 48 h were increased, while 329 transcripts at 6 h and 2382 transcripts at 48 h were decreased (Fig. 3*A*). After CD1530 treatment of PAR cells, 184 transcripts at 6 h and 2190 transcripts at 48 h were upregulated, while 67 transcripts at 6 h and 2044 transcripts at 48 h were downregulated (Fig. 3*B*).

We next performed gene set enrichment analysis (GSEA) (34) on the 6 h and 48 h treatment groups (Fig. 3, *C* and *D*), as well as ORA (Supporting Information Fig. S2) on differentially expressed genes with p-adj. < 0.01 and |log2(FC)| > 0.3. At 6 h, genes involved in epithelial cell proliferation were downregulated in both RA and CD1530 (Fig. 3, *C* and *D*) treated PAR cells, with an early enrichment of genes upregulated in apoptotic and antiproliferative processes (Supporting Information Fig. S2). At 48 h, upregulated gene sets from both RA and CD1530 treatments were primarily enriched for genes involved in oxidative phosphorylation and cellular respiration in PAR cells (Fig. 3, *C* and *D*).

**Figure 4:**
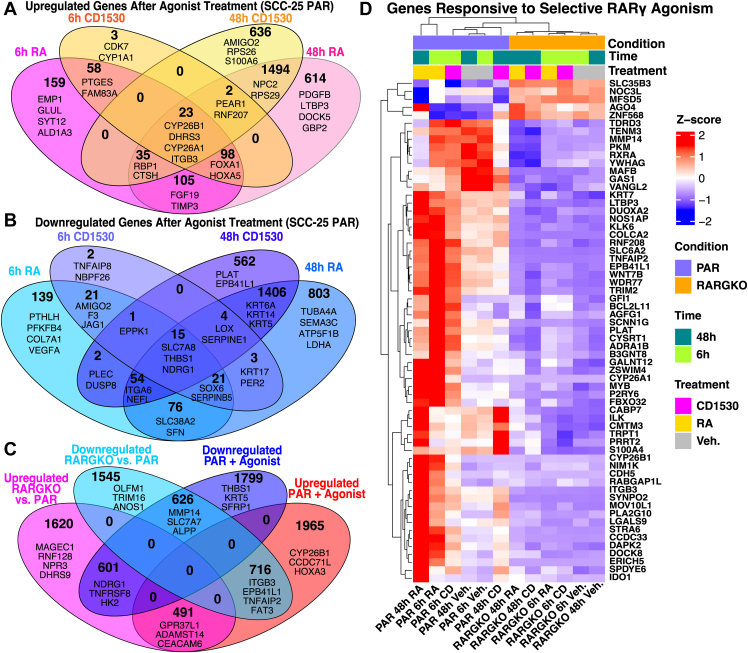
**Comparison of gene responses to agonist treatments**. *A* and *B*, Venn diagrams of genes upregulated (A) and downregulated (B) after 6 h and 48 h with either CD1530 or RA (p.adj < 0.01, |log_2_FC| > 0.3). *Top* 2 to 4 genes in each group by stat are shown as examples. *C*, Venn diagram of genes with altered expression in response to RARG deletion and/or agonism (p.adj < 0.01, |log_2_FC| > 0.3). *Top* 2 to 4 genes in each group by stat are shown as examples. Gene set lists for *panels A*–*C* are in Supporting Information Data S1; *D*, Clustered heatmap identifying relative expression changes among genes that robustly respond to RAR agonism only in the presence of functional RARγ. RAR, retinoic acid receptor.

Thus, a subset of the transcripts regulated by RA in the SCC-25 PAR cells was regulated by CD1530, as expected, and these data suggest that at 6 h about a third of the total transcripts increased by RA involved actions of RARγ because these transcripts were also increased by CD1530 (Fig. 3, *A* and *B*, 4*A*), including genes that are well studied in other cell types that respond to RA, such as HOXA3, CYP26A1, and DHRS3 (35), as well as several keratinocyte-related genes (Fig. 3, *A* and *C*, RA; B and D, CD1530). Only three mRNAs were significantly upregulated by CD1530 at 6 h but not by RA at either time point (Fig. 3*A*), and similarly only NBPF26 and TNFAIP8 were significantly downregulated at 6 h only by CD1530 (Fig. 3*B*). Among the transcripts reduced in response to RA at 6 h, 58 were also reduced in response to CD1530 at 6h, including THBS1, CDKN1C (Fig. 3, *A* and *B*), JAG1, NDRG1, and SOX6 (Fig. 4*B*). Several cytokeratin transcripts, including KRT6A, KRT14, and KRT5, were downregulated at later time points after both agonist treatments.

### Comparison of transcripts and signaling pathways in SCC-25 PAR and SCC-25 RARGKO cells in the presence of agonists

We examined the gene overlap in response to both RA and CD1530 treatments to identify genes whose transcripts changed were either attributable to RARγ actions or potentially associated with actions of other RARs (α, β). A significant number of mRNAs was altered in untreated RARGKO compared to PAR and did not respond to treatment with either RA or CD1530 at 6 and 48 h in PAR (Fig. 4*C*). Several of the top overexpressed transcripts in RARGKO that did not respond to either agonist treatment in PAR included DHRS9 and RNF128 (GRAIL), as well as members of the Melanoma Antigen Gene (MAGE) (Fig. 4*B*) and SPRR (small proline-rich) families (Supporting Information Data S1); transcripts that were constitutively downregulated in RARGKO and unresponsive to agonist in PAR included OLFM1, TRIM16 (Fig. 4*C*), IRX1, FAM50, and KRT4 (Supporting Information Data S1). These genes might reflect ligand-independent and/or nonnuclear functions of RARγ in supporting other signaling pathways, some of which have been characterized in other cell types. For example, RARγ has been shown to physically complex with RIPK1 during DNA-damage induced initiation of necroptosis in mouse embryo fibroblasts and recently was shown to associate with the inflammasome adapter ASC to mediate pyroptosis in immune cells (27, 36). We also discovered some genes that were neither responsive to CD1530 nor RA in the RARGKO (Fig. 4*D*), suggesting that RARγ is the primary RAR responsible for modulating their transcription in SCC-25. Examples include MAFB, CYB26B1, ERICH5, and ITGB3.

### CUT&RUN (C&R) maps RARγ genomic binding in SCC-25 PAR cells

To identify direct gene targets of RARγ and identify regulatory binding sites across the genome, we extracted nuclei from SCC-25 PAR and RARGKO cells and performed CUT&RUN in these samples using antibodies to RARγ, histone H3 lysine 4 trimethyl (H3K4me3), and histone 3 lysine 27 trimethyl (H3K27me3); immunoglobulin G (IgG) was a negative control (Fig. S1, *C* and *D*). H3K4me3 is an established marker of active transcription (37) and changes in H3K4me3 breadth affect cell identity and transcriptional consistency (38), while H3K27me3 is often studied as an indicator of facultative heterochromatin (39, 40, 41).

**Figure 5:**
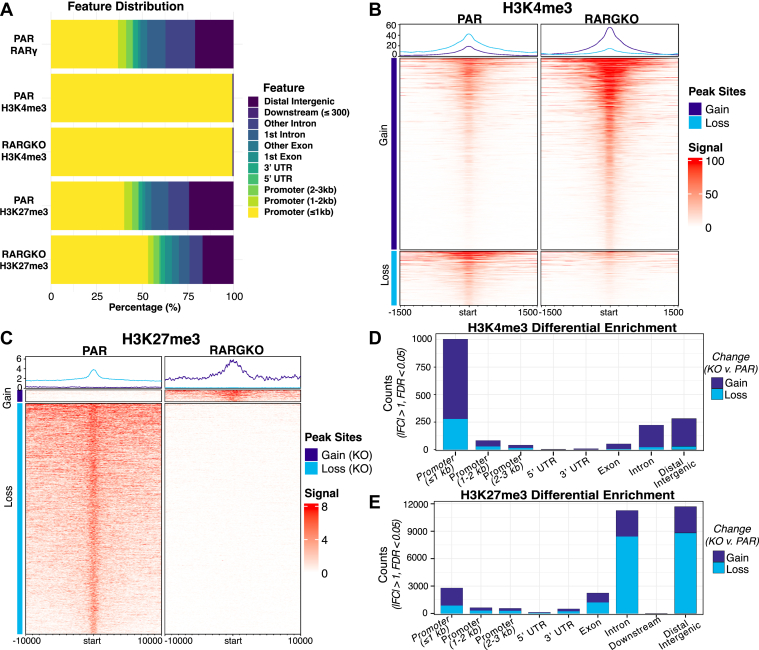
**CUT&RUN analysis in SCC-25 cells maps constitutive binding sites for RARγ and identifies chromatin accessibility changes in RARGKO cells**. PAR and RARGKO cells were seeded in triplicates, gently fixed with 0.1% formaldehyde, and nuclei were extracted for CUT&RUN targeting RARγ, H3K4me3, and H3K27me3, using IgG as a negative control. *A*, genome-wide profiling of H3K27me3, H3K4me3, and RARγ peaks by region in relation to identifying percent distribution within regulatory regions in relation to their distance from a gene TSS. *B*, DiffBind profiling of H3K4me3 peaks and *C*, H3K27me3 peaks. Sites are clustered by gain or loss of signal in RARGKO *versus* PAR, and signal intensity is depicted from *white* (0) to *dark pink* (max, PAR) or *dark purple* (max, RARGKO). Broader H3K27me3 peak signal is depicted across 20 kb and narrower H3K4me3 signal is depicted across 3 kb. *D* and *E*, genome annotation for differentially bound H3K4me3 (*D*) and H3K27me3 (*E*) sites, with peak counts for sites gained/lost in RARGKO stacked. CUT&RUN, cleavage under targets and release using nuclease; IgG, immunoglobulin G; RAR, retinoic acid receptor; TSS, transcription start site.

We performed peak calling using MACS3 (42) to obtain peak sets and maximum signal regions for each target, and replicates were overlayed for peak visualization. H3K4me3 was consistently enriched near transcription start sites (TSSs), and H3K27me3 localization among top peaks indicated a general shift in H3K27me3 toward promoter regions in RARGKO compared to PAR cells (Fig. 5*A*). RARγ tended to be associated with Cis-regulatory element regions (TSS ± 3000 bp), and approximately 38% of RARγ peak summits mapped to proximal promoter regions (TSS ± 250 bp) (43, 44). We also found that nearly 20% of RARγ peaks were in distal intergenic regions, suggesting association with both *cis*-regulatory elements and *trans*-acting factors (Fig. 5*A*). We identified RARγ peak sites in all chromosomes, but areas of clustering could be found in some chromosomes, particularly chr1 and chr12 (Supporting Information Fig. S3*A*). Chromosome 12 contains the human RARγ gene locus and several families of genes expressed abundantly in squamous epithelial cells, such as the KRT genes that encode for keratin proteins. Genome coverage plotting for RARγ localization further showed absent or markedly reduced signal from RARGKO cells (Supporting Information Fig. S3*A*), indicating that the RARγ antibody was specific for RARγ, and this enabled peak analysis at areas of low enrichment.

### Global chromatin landscape alterations in response to loss of RARγ

Because RARγ associates with a variety of genomic regions and the robust RARγ peaks we identified reflect less than 10% of the differentially expressed genes between PAR and RARGKO (Table 1), we next examined whether RARGKO cells have broadly different chromatin architecture. We ran differential enrichment analysis with DiffBind on MACS3 narrow peaks called for H3K4me3 (Fig. 5, *B* and *D*, Supporting Information Fig. S3, *B* and *D*) and on broad peaks called for H3K27me3 (Fig. 5, *C* and *E*, Supporting Information Fig. S3, *C* and *E*) to assess genome-wide changes in H3K4me3 and H3K27me3 levels between PAR and RARGKO. H3K27me3 and H3K4me3 consensus peak sets were identified as regions with signal in at least two replicates. Of the 94,290 consensus H3K4me3 peaks, 9291 sites were differentially enriched with false discovery rate (FDR) < 0.05 between RARGKO and PAR, and 7278 (78%) of these sites showed an enriched signal in RARGKO. Most of the sites with more pronounced changes in H3K4me3 signal also appeared to coincide with increases in peak breadth (Fig. 5, *B* and *D*), suggesting changes at those sites to transcriptional consistency (38). In contrast, of the 110,515 consensus H3K27me3 peaks, 53,743 H3K27me3 peak sites had differential enrichment (FDR < 0.05) between RARGKO and PAR, of which 54% were depleted in RARGKO. However, for sites with more dramatic changes in signal (|Log_2_FC| > 1), 20,194 regions had reduced H3K27me3 in RARGKO compared to 9577 regions with increased H3K27me3 (Fig. 5, *C* and *E*). Promoter regions generally were sites of increased H3K27me3 deposition in RARGKO, while differential H3K27me3 signal at distal intergenic and intronic regions tended to be depleted in RARGKO (Fig. 5*E*).

**Table 1:** Intersection of genes associated with RARγ peaks in SCC-25 PAR and differentially expressed genes (DEG) from RNA-seq identifies putative direct RARγ transcriptional targets

Time	Differentially expressed after treatment with:	Transcript changes in PAR	Treatment response in KO	Total # DEG	# DEG w/RARγ peaks	# DEG w/RARγ at CRE (<3000 bp)	# DEG w/RARγ at Prox. Prom. (<250 bp)
6 h	CD1530 or RA	Reduced	Yes	18	7	4^a^	3^a^
			No	49	14	11^b^	6^b^
	CD1530 only		Yes	2	1	1	1
			No	14	6	3	2
	RA only		Yes	51	12	7	2
			No	291	67	41	28
	CD1530 or RA	Increased	Yes	85	41	22^a^	18^a^
			No	124	53	33^b^	21^b^
	CD1530 only		Yes	3	0	0	0
			No	9	2	2	1
	RA only		Yes	40	16	10	9
			No	277	84	54	39
48 h	CD1530 or RA	Reduced	Yes	1347	265	178^a^	141^a^
			No	198	38	27^b^	20^b^
	CD1530 only		Yes	466	117	67	48
			No	101	24	18	17
	RA only		Yes	312	57	39	27
			No	655	133	80	65
	CD1530 or RA	Increased	Yes	1610	213	160^a^	146^a^
			No	221	30	16^b^	11^b^
	CD1530 only		Yes	545	72	62	57
			No	157	15	10	10
	RA only		Yes	328	81	52	39
			No	671	97	73	55

DEGs (p.adj < 0.05, |Log2(FC)| > 0.3) are categorized by (1) changes in expression at earlier or later time points, (2) how transcription changes for PAR + CD1530/RA *versus* PAR + vehicle alone, (3) whether the genes do not similarly respond to treatment in RARGKO cells, and (4) whether they are within the set of RARγ binding sites (-log(q) > 5) in PAR cells. Peak/DEG overlap is further subdivided to identify DEGs with RARγ binding at a cis-regulatory region within 3 kb of the gene’s TSS, and at proximal promoter regions within 250 bp of the TSS. Peak and DESeq2 data for the table contents are in Supporting Information Data S6.

a Putative direct RARγ targets that can also be modulated by other ligand-bound tsype II NRs.

b Putative direct and isotype-specific RARγ targets.

We performed analysis of motif enrichment (AME) (45, 46) on top differentially enriched H3K4me3 sites and identified motifs for NANOG (NANOG.H12CORE.0.P.B), SOX2 (SOX2.H12CORE. 1.P.B), and POU5F1 (POU5F1.H12CORE.1.P.B) as significantly enriched in RARGKO over PAR (Supporting Information Fig. S4*A*). These three transcription factors are core transcriptional drivers of pluripotency factors. At the mRNA level, comparing RARGKO and PAR, neither NANOG nor POU5F1 was significantly altered, and SOX2 was upregulated in RARGKO (log_2_FC = 0.37, p.adj < 0.00001 (Supporting Information Data S1). Overall, these data suggest that RARγ counters a stem-like phenotype in the SCC-25 PAR cells.

Genome Ontology enrichment analysis with HOMER (47) v5.1 tools also revealed that there is a significant, global increase in both H3K27me3 and H3K4me3 marks in the RARGKO *versus* PAR cells at CpG islands (CGI) (Supporting Information Fig. S4, *B* and *C*). The presence of H3K4me3 at these regions suggests that they are areas of active transcription, while the global spread of H3K27me3 in CpG islands suggests that there are, paradoxically, also histone modifications central to facultative heterochromatin formation (41). Some examples of genes at which this increase in H3K27me3 occurs at the CpG islands are FOXF1, FOXF2, DUSP9, and CYP26B1 (Supporting Information Data S3). This feature may have relevance to head and neck squamous cell carcinoma (HNSCC) in that it has been reported that an aberrant, global increase in H3K27me3 marks around promoter regions of these genes occurs in other cancers, such as gastric cancer (48). In some cases, such as for DAB2IP, a decrease in promoter H3K4me3 corresponded to an increase in adjacent H3K27me3, whereas at other genes, such as FAT3, new H3K27me3 signal spread directly across regions where H3K4me3 was lost. In other cases, bivalency was newly established, such as with SFRP1, or lost (Supporting Information Fig. S4*D*). SFRP1 (Secreted frizzled-related protein 1), is a Wnt antagonist previously found to have a bivalent promoter that undergoes transcriptional switching during embryonic stem cell differentiation (49).

### RARγ binds to regulatory regions of genes associated with apical junction adhesion, extracellular matrix structure, and squamous epithelial stratification

**Figure 6:**
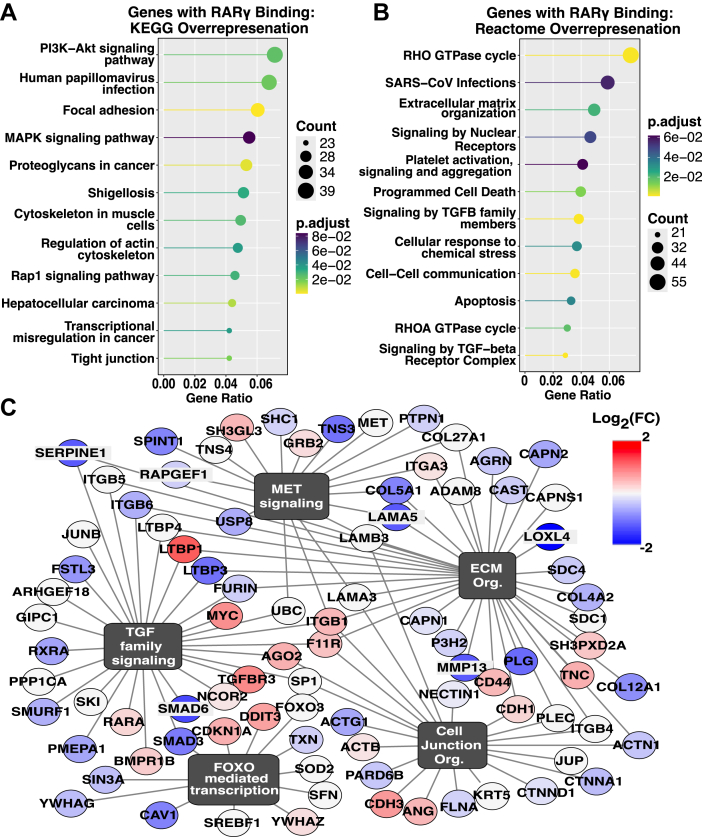
**Analysis of CUT&RUN generated RARγ peaks identifies putative gene targets for transcriptional modulation in SCC-25 cells**. ClusterProfiler dot plots of gene set overrepresentation analysis on the *top* 2000 MACS3-called RARγ peaks by integer score (1141 unique gene IDs) using *A*, reactome and *B*, KEGG databases. *C*, network plot of enriched pathways among genes associated with RARγ peaks and corresponding differential expression of these genes in RARGKO *versus* PAR. CUT&RUN, cleavage under targets and release using nuclease; KEGG, Kyoto Encyclopedia of Genes and Genomes; RAR, retinoic acid receptor.

**Figure 7:**
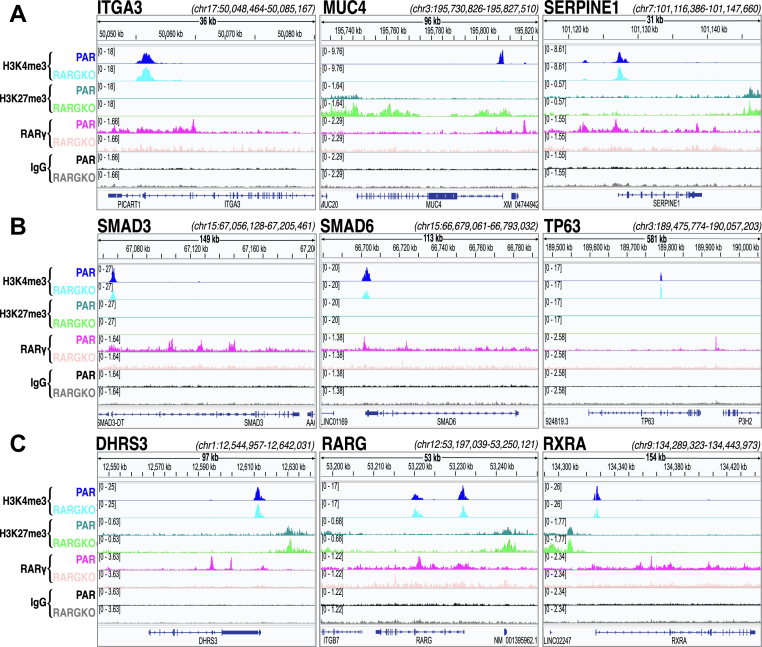
**Visualization of RARγ, H3K4me3, and H3K27me3 signal at select gene loci**. *A*, *B*, and *C*, IGV tracks of *Escherichia coli*-normalized CUT&RUN alignments to hg38 genome for both PAR (*darker blue*, *darker green*, *magenta*, and *black*) and RARGKO (*cyan*, *light green*, *light pink*, and *gray*) samples. Replicates are overlaid for each group, and RefSeq gene exons are mapped at the *bottom*. Representative samples shown for genes involved in (*A*) ECM maintenance and adhesion, (*B*) Epithelial cell differentiation, and (*C*) retinoid signaling and metabolism. CUT&RUN, cleavage under targets and release using nuclease; ECM, extracellular matrix; RAR, retinoic acid receptor.

We identified approximately 6000 RARγ peaks in SCC-25 PAR (-log_10_q > 5 and no enrichment in RARGKO samples). Pathway analysis of genes annotated to these peaks shows enrichment of pathways that regulate cell proliferation and apoptosis, such as the PI3K-Akt and MAPK axes, as well as enrichment of genes involved in TGFβ/SMAD signaling and focal adhesion/extracellular matrix interaction (Fig. 6, *A* and *B*). We found robust RARγ C&R signals at the extracellular matrix related genes ITGA3, SERPINE1, and MUC4 (Fig. 7*A*), MMP7, MMP13, MMP14, ITGB3, ITGB6, ITGA6, ITGA3, COL7A1, COL12A2, and COL4A2 (Supporting Information Data S2). These genes were also differentially expressed between PAR and RARGKO (Fig. 6*C*).

In some cases, such as in SERPINE1 and ITGA3, the H3K27me3 and H3K4me3 landscape was not appreciably altered between RARGKO and PAR; however, for the MUC4 promoter region, the H3K4me3 signals, both the upstream and overlapping RARγ signals in PAR, were replaced with H3K27me3 accumulation in RARGKO (Fig. 7*A*). Epithelial differentiation-related genes SMAD3, SMAD6, and TP63 (Fig. 7*B*) displayed RARγ peaks at both proximal TSS regions, such as in SMAD3, and at distal regions > 3000 bp away from the TSS, such as in TP63. We also identified an enrichment of SMAD3 and SMAD4 DNA binding motifs near RARγ peak summits (Supporting Information Fig. S4*F*), suggesting that RARγ may modulate TGFβ signaling through both direct transcriptional regulation of downstream effectors and colocalization at gene target regulatory regions. Interestingly, transcripts for both regulatory Smad3 and inhibitory Smad6 transcripts were reduced more than twofold in RARGKO *versus* PAR, in line with a reduction in the H3K4me3 signals in the promoters of both SMAD3 and 6 (Fig. 7*B*). The H3K4me3 signal for TP63 was localized entirely at its internal promoter that drives transcription of the ΔNp63 isoform, which lacks the N terminal activating domain of full-length p63, in both PAR and RARGKO SCC-25 cells (Fig. 7*B*) (50).

Subsequently, 35% of the top 2000 RARγ peaks (by integer score) mapped to 432 genes that were differentially expressed in RARGKO *versus* PAR from RNA-seq analysis (Supporting Information Data S1, S2). GSEA of the top peaks showed pathway overlap between RARγ peaks and gene sets upregulated and downregulated upon loss of RARγ (Figs. 6*C* and 2, *C* and *D*). Genes involved in extracellular matrix organization and TGFβ-signaling are changed (Fig. 6*C*), indicating a role for RARγ in TGFβ signaling, which is often dysregulated in OCSCC development (51). We found RARγ peaks in many genes associated with cell adhesion and differentiation and some were also differentially expressed in RARGKO *versus* PAR (Fig. 2, *A* and *F*; Supporting Information Data S1).

### RARγ binds at regulatory regions for other type II nuclear receptors and genes involved in retinol metabolism

We identified several RARγ peaks at the promoter and intronic regions of the primary RARγ heterodimerization partner, RXRA. Loss of RARγ coincided with a marked reduction in H3K4me3 signal at the RXRA promoter (Fig. 7*C*). We observed that RXRA mRNA levels were similarly reduced by more than 30% (p-adj. = 6.5E-115) (Supporting Information Data S1) in RARGKO cells. RARγ also associated with its own promoter regions (Fig. 7*C*), as well as displaying weaker associations with the promoters of RARA and RARB (Supporting Information Data S2), suggesting that RARγ may be directly involved in modulating transcription of RARs and their interactors in response to RA. Although RARβ has an established RARE within its own promoter region (52), the potential for RARγ to facilitate its own transcription in response to RA availability has not been well characterized. RXRα also dimerizes with type II nuclear receptors such as peroxisome proliferator-activated receptor gamma (PPARγ) and Vitamin D receptor (VDR) (53) and RARγ binding at both RXRα and a downstream intergenic site near the PPARγ locus (Supporting Information Data S2) suggests that alterations to RARγ signaling functions can impact genomic signaling by other type II nuclear receptors *via* direct transcriptional modulation or through modulating the availability of RXRα as a dimerization partner.

We further identified RARγ peaks associated with genes involved in retinol metabolism, including DHRS3 (Fig. 7*C*). DHRS3 provides critical negative feedback regulation in response to VA signaling by reducing the bioavailability of its metabolites *via* converting retinaldehyde back to retinol (54, 55, 56). Taken together, our data suggest that RARγ modulates nuclear receptor signaling more globally because of RARγ binding to the promoters of multiple nuclear receptor binding partners and retinol metabolizing genes.

### Integration of RNA-seq analysis from agonist treatments and CUT&RUN analysis of RARγ peak sites reveals ligand-bound RARγ-specific genomic target motifs

**Figure 8:**
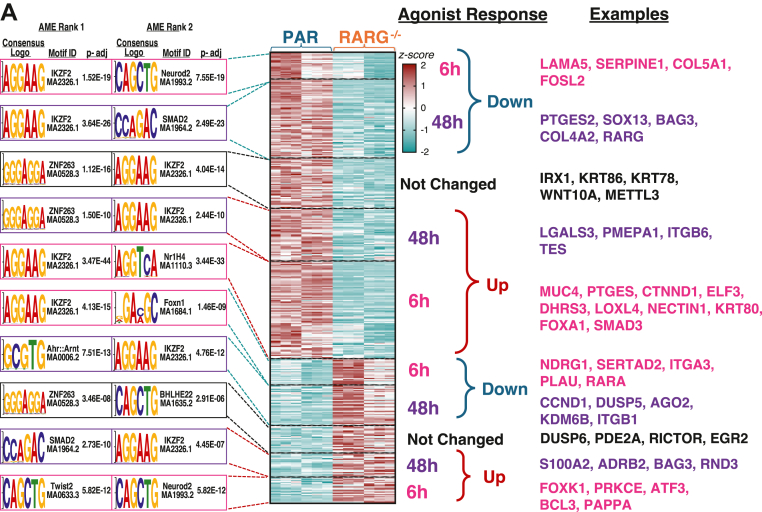
**Integration of CUT&RUN RARγ peak sites and gene responses to changes in RARγ signaling reveals differences in transcriptional regulation with and without added ligands**. *A*, heatmap of differentially expressed genes between PAR and RARGKO (|log_2_FC| > 0.3 and p.adj < 0.05) with one or more RARγ binding sites. Columns reflect z-scores for each vehicle-treated sample for each genotype condition (PAR and KO). Genes are clustered by direction of transcriptional response to RARγ agonism in PAR (CD1530 and/or RA) and by whether a significant response is observed at 6 h or only at 48 h (p.adj < 0.05). *Top* two differential motif enrichment results from AME analysis (Fisher’s test) are shown for each subset compared to all other RARγ CUT&RUN binding sites. AME, analysis of motif enrichment; CUT&RUN, cleavage under targets and release using nuclease; RAR, retinoic acid receptor.

We next focused on differentially expressed genes between PAR and RARGKO that were associated with RARγ peaks in our CUT&RUN analysis to examine the primary transcriptional outcomes of RARγ localization to specific *cis*-regulatory regions (Fig. 8). Approximately half of the genes at which RARγ was associated by CUT&RUN in PAR that were also downregulated in RARGKO compared to PAR by RNA-seq were upregulated after agonist treatment in PAR; these include SMAD3, FOXA1, ELF3, KRT80, PTGES, and CTNND1 at 6 h. Of the genes at which RARγ was associated by CUT&RUN in PAR and that were also upregulated in RARGKO compared to PAR by RNA-seq, approximately half were downregulated after agonist treatment in PAR, including NDRG1, ITGA1, and PLAU, Interestingly, some RARγ peaks were in the regulatory regions of genes that were transcriptionally unresponsive to agonist treatment but were differentially expressed between PAR and RARGKO. Among these, expression of IRX1, WNT10A, KRT78, KRT86, NRL, SLC2A6, SLCO3A1, SUSD1, and TAPBP was decreased more than two-fold in RARGKO, while expression of DUSP6, EGR2, RICTOR, and PDE2A increased more than two-fold in the absence of RARγ (Fig. 8). These data suggest that RARγ also modulates chromatin accessibility and gene transcription without ligand binding.

We defined direct ligand-bound RARγ transcriptional targets as the intersection of (1) genes with RARγ peaks at promoter and/or enhancer regions from our CUT&RUN of PAR cells (with emphasis on proximal promoter regions ≤ 250 bp from the TSS); and (2) genes that were differentially expressed in response to agonist treatments in PAR cells. Among the ligand-dependent RARγ targets, there was a subset of genes responsive to RA treatment in both PAR and RARGKO cells that was also associated with RARγ peaks, and these could be genes that are also targets of RARα and/or β (Table 1, Supporting Information Data S6). These genes include PTGES, CYP26B1, DHRS3, RBP1, HOXA4, NR2F1, KRT7, KRT15, KRT80, and ELF3 among genes upregulated in response to RA treatment, and CCNA1, THBS1, EGR1, KRT5, and LAMB3 among genes downregulated in response to RA treatment (Supporting Information Data S6).

We identified CNR1, CEL, CYSRT1, IRF1, GPR37L1, MUC4, PPARG, NAV2, SMAD3, and SMAD6 as genes directly and primarily upregulated by ligand-bound RARγ. These genes had RARγ peak sites in PAR, and were also both downregulated in RARGKO cells and unresponsive to either agonist treatment in these KO cells (Fig. 8, Supporting Information Data S6).For example, MUC4 was virtually silenced (nTPM < 10 for all) in RARGKO, but was expressed in PAR (nTPM > 250, log_2_(FC) = 7.6) and upregulated upon CD15390 and RA treatments in PAR, indicating early and sustained mRNA upregulation in response to agonist treatment in PAR. CEL, CYSRT1, and MUC4 transcripts were markedly reduced in RARGKO, compared to PAR, without addition of ligand but had strong RARγ binding signal at their regulatory elements (Supporting Information Data S2), suggesting that RARγ binding in the absence of added agonist promotes their expression. We similarly identified AMIGO2, JAG1, and STK17B as genes directly repressed by ligand-bound RARγ rather than RARα/β. Several AP-1 factors, including FOS, JUN, and JUNB, had RARγ binding sites at their promoters and were also downregulated upon agonist treatment in PAR, but their transcription was not significantly altered in RARGKO compared to PAR.

A subset of the RARγ binding sites overlapped with super enhancer regions identified in SCC-25 cells (57). We compared the RARγ CUT&RUN peak sites with these enhancer regions and found RARγ bound specifically at a super enhancer site targeting the HOX gene cluster, as well as other sites targeting KRT gene clusters (Supporting Information Data S4). These results indicate that RARγ in SCC-25 cells may regulate transcription of multiple epithelial differentiation gene clusters in a coordinated manner through its binding at super enhancer sites.

### Differential enrichment of spaced repeats in RARγ peaks between genes that respond differently to ligand treatment

**Figure 9:**
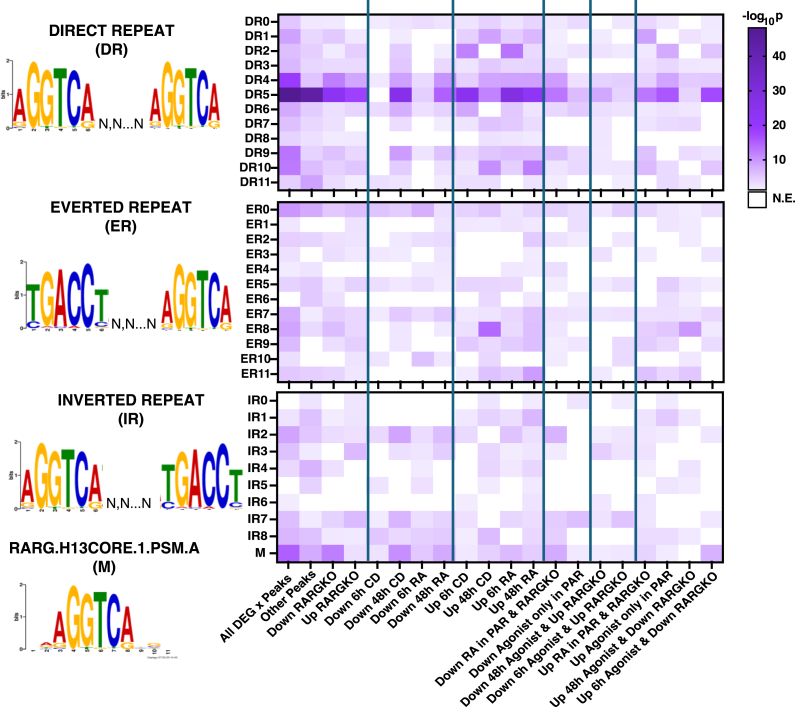
**Characterization of repeat-type enrichment in RARγ peaks**. Repeat-type enrichment in RARγ peaks with q < 0.00001 in SCC-25 PAR, according to early and late transcriptional responses to agonist treatment at associated genes (p.adj < 0.05, |log_2_(FC)|>0) as well as the presence or absence of functional RARγ. Values in *white* reflect motifs that are not enriched (-log_10_*p* < 1.3, N.E.). RAR, retinoic acid receptor.

Previously characterized binding motifs for the RARs include a *5′*-RGKTCA-*3′* half site, (where R is A/G and K is G/C) separated in direct repeats (DRs) by 0, 1, 2, or 5 spacer nucleotides (58, 59). Studies have identified other sites, such as an everted repeat (ER8) site (60) and inverted repeat (IR) binding sites (59). We identified differences in the binding motifs among genes differentially expressed plus *versus* minus agonist and in PAR *versus* RAGKO cells (Fig. 9).

Subgrouping the RARγ peaks by associated gene responses to RARγ functional alterations, we then tested the enrichment of 5′RGKTCA direct, inverted, and everted repeats with spacers ranging from 0 to 12 nucleotides (Fig. 9). As expected, we identified numerous DR5 motifs at sites of maximum RARγ binding signal, particularly in peaks associated with genes that were upregulated in response to RARγ agonism, indicating DR5 motifs are one of the major RARγ binding sites to promote gene expression.

Notably, genes with RARγ peaks that were downregulated upon CD1530 or RA treatment did not show relatively DR5 rich elements, but rather tended toward ER0 sites; in contrast, the genes upregulated upon CD1530 or RA addition were enriched for DR6 and DR2 sites. Conversely, substantively less enrichment of RARγ peaks was found on these sites in genes identified in our RNA-seq as late-response (48 h) genes. The peak set mapping to genes that only responded to agonist treatment when RARγ was present had a higher relative enrichment for IR7 elements among downregulated genes, and IR1 elements among upregulated genes. Many RARγ peak centers were enriched in a monomeric half site that may indicate loose association with chromatin in the absence of RXRA rather than direct binding as a heterodimer.

**Figure 10:**
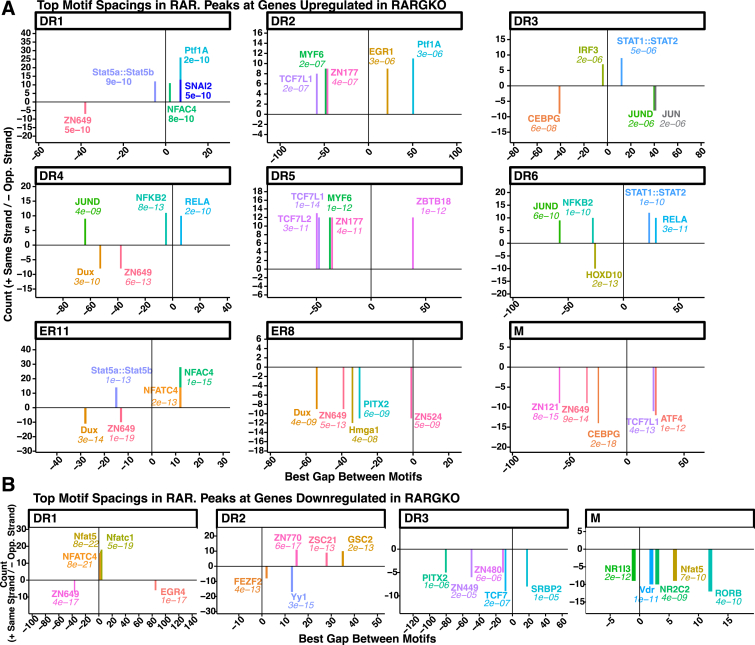
**Μotif co-occurrence at RARγ peak sites**. *A* and *B*, *top* significantly enriched spacings (labeled by motif-associated factor and E-value) identified with SpaMo between primary RARγ 3’RGKTCR’ hexameric half-site motif and secondary nonredundant HOCOMOCO/JASPAR motifs within peaks associated with genes upregulated (*A*) or downregulated (*B*) in RARGKO *versus* PAR (p.adj < 0.05, |log_2_FC|> 0.3). Positive *y*-axis values identify occurrences of the secondary motif on the same strand as RARγ half-site, while negative *y*-axis values identify opposite strand occurrences. Positive *x*-axis values identify downstream (3′) occurrences and negative *x*-axis values identify upstream (5′) occurrences. SpaMo calculates E-values by identifying enriched spacings between the input primary motifs and secondary motifs using a binomial test, adjusting for different numbers of spacings and orientations, and for the number of secondary motifs. RAR, retinoic acid receptor.

To characterize the nucleotide sequences surrounding RARγ binding sites at motifs with different gapped-lengths, we ran SpaMo (61) analysis to identify significantly enriched spacings between the RARγ motifs identified within the PAR CUT&RUN peaks and secondary motifs within the nonredundant HOCOMOCO and JASPAR vertebrate databases (Fig. 10). For this analysis, we focused on repeat types that had existing biological evidence as functional RAREs. Recent work by Bhimsaria *et al*. addressed prior issues in identifying nuclear receptor heterodimer binding sites that often vary in spacing between half-site repeats (59). We validated their motif-finding workflow (MinSeq) using their publicly available scripts and SELEX data, enabling us to generate a set of position-weighted matrices for RARγ binding (combining SELEX data with RARγ alone, RARγ bound to RA, and RARγ bound to RXRA) and distinguish the known DR, ER, IR, and monomer (M) motifs. Among peaks annotated to genes both upregulated and downregulated in RARGKO *versus* PAR, we identified differences in transcription factor colocalization depending on repeat type. Motifs for several immune-related transcription factors, including NF-κB, STAT1, EGR1, STAT5 and Nfat family proteins, were enriched at recurring spaced intervals from RARγ binding sites among genes upregulated in RARGKO (Fig. 10*A*), with NFAT factor binding motifs also enriched near DR1 sites at genes downregulated in RARGKO (Fig. 10*B*). Motifs associated with cancer stem cell phenotype drivers were enriched at RARγ peaks associated with both upregulated and downregulated genes, such as Slug (SNAI2) (Fig. 10*A*) and YY1 (62) respectively (Fig. 10*B*).

### Motif analysis reveals co-occurrence of RARγ occupancy and known JUN/FOS motifs

Motif enrichment analysis was performed on the top 2000 RARγ peaks (by MACS3 integer score with no enrichment detected in RARGKO – RARγ peaks) using HOMER (47) command line tools to identify known and *de novo* motifs associated with these peaks (Supporting Information Fig. S5, *A* and *B*). We found that 22% of the top peaks contained an AP-1 like motif containing the consensus sequence 5′-TGA(G/C)TCA-3′ recognized by most activated Jun and Fos family members (63). In terms of transcript levels, AP-1 sites were enriched in the promoter regions of gene sites that were downregulated early in response to RARγ agonism, (Supporting Information Fig. S5*C*), but were present among genes that were both upregulated and downregulated in PAR *versus* RARGKO.

## Discussion

We identified RARγ-mediated signaling as an important regulator of the transcriptional landscape, with significant implications for epithelial differentiation, adhesion, and OCSCC progression. By integrating transcriptomic and epigenomic genome-wide approaches, we discovered direct RARγ target genes, defined as genes where RARγ is bound, involved in transcriptional programs crucial for squamous epithelial cell identity and for cell differentiation. We also elucidated RARγ′s influence on chromatin accessibility, highlighting RARγ′s dual roles in transcriptional activation and repression.

Notably, transcripts for some genes associated with stratified squamous cell differentiation, including NOTCH1, NOTCH3, and the Notch ligands JAG2 and DLL1, were downregulated in RARGKO without added ligand (Fig. 2*E*), consistent with previous reports that Notch signaling is essential for maintaining epithelial stratification and that Notch signaling regulates cell fate decisions (8, 9). Thus, our data indicate that RARγ contributes to signaling through the Notch pathway and are consistent with the report that loss of expression and/or mutation of the tumor suppressor NOTCH1 or Notch signaling pathway genes account for ∼67% of human HNSCC cases that include OCSCC (23). We also observed RARγ peak enrichment at the JAG1 promoter, with JAG1 both specifically downregulated in response to RARγ agonism (Fig. 4*B*, Supporting Information Data S1, S6) and constitutively upregulated in RARGKO *versus* PAR (Fig. 2*E*). JAGs are Notch ligands that, when mutated in HNSCC, can cause HNSCC development (23). Furthermore, the intracellular domain of JAG1 (JICD1) promotes tumorigenesis independent of Notch signaling through activating Sox2 transcriptional expression (64). Thus, our data suggest RARγ may be a negative regulator of Jag1, but that, conversely, RARγ increases expression of the other Notch ligands and receptors.

Through genome-wide transcriptomics analysis, we also identified a substantial number of differentially expressed genes involved in epithelial–mesenchymal transition, development, and immune response pathways (Fig. 2, *C* and *D*), Our data suggest that RARγ may play a role in maintaining tight cross-regulation between key differentiation and development pathways in the squamous epithelium.

By comparing responses to RA and the RARγ-selective agonist CD1530 we could distinguish between RARγ transcriptional effects and those likely mediated by other RAR isotypes (*e*.*g*. RARs α and β). Both RA and CD1530 induced well-known RA-responsive genes, such as CYP26A1 and HOXA1 (35), in PAR cells. However, mRNAs for genes including ITGB3, ERICH5, CYP26B1, and WNT7B were only upregulated in response to agonist treatment in PAR cells, but not in RARGKO cells treated with either RA or CD1530; these results show that these are RARγ gene targets in SCC-25 cells that are activated in a ligand-dependent manner specifically through the RARγ isotype (Fig. 4*D*). The intersection of genes associated with RARγ peaks identified through CUT&RUN and DEGs in response to both the loss of RARγ and treatment with RARγ agonists further identifies candidate RARγ target genes that respond to RARγ signaling in a ligand-dependent or independent manner (Fig. 8). Silencing of MUC4 expression in RARGKO cells, despite its robust upregulation by CD1530 and RA in PAR cells, indicates that RARγ directly activates MUC4 transcription (Figs. 2*B* and 4*C*). Indeed, Figure 7*A* shows that RARγ directly binds to the MUC4 promoter region, with H3K27me3 accumulation in the same promoter region in RARGKO SCC-25 cells. Notably, MUC4 is implicated in cell differentiation in stratified squamous epithelial tissues such as the esophagus and tongue (65). Conversely, genes such as PPARGC1A, IRX1, and KRT4, were largely silenced in RARGKO (Fig. 2*B*), but were unresponsive to agonist treatments in PAR cells and did not have RARγ peaks near their *cis*-regulatory regions, suggesting that RARγ may regulate expression of these genes indirectly in a ligand-independent manner.

Motif analysis of RARγ peak sites has not been reported to date. Genomic analysis with the nuclear receptor LXR identified distinct motifs associated with agonist mediated repression *versus* activation and other work identified unique binding sites for LXR isotypes α and β (66, 67).

Our CUT&RUN analysis provides clear evidence in PAR SCC-25 cells of RARγ occupancy at genomic DNA regulatory elements, with approximately 20% of RARγ binding sites located in distal intergenic regions; these data suggest roles in enhancer-mediated transcriptional regulation (Fig. 5*A*). Approximately 50% of RARγ binding sites were in *cis*-regulatory regions, with most of these sites less than 1000 bp from the TSSs. Notably, RARγ peaks were highly enriched near genes involved in cell surface signaling and adhesion, including SERPINE1, ITGB3, ITGA6, and COL4A2, which are known to control both epithelial plasticity and tumor progression (26, 68, 69).

In addition, differential histone modification analysis revealed a significant shift in H3K27me3 deposition in RARGKO cells without agonist treatment into promoter regions, indicating a spread of facultative heterochromatin, but there were also bivalent sites (sites with two opposing regulatory marks) in some genes in the RARGKO cells (Supporting Information Fig. S4*D*). The overlap of H3K4me3 and H3K27me3 at gene promoters in embryonic stem cells is particularly common, and such bivalency is proposed to maintain repressed genes in a “poised” state for rapid activation (70, 71). The overall global increase in both H3K4me3 and H3K27me3 marks in RARGKO across CpG islands and the dramatic reduction of H3K27me3 at sites of enrichment in PAR *versus* RARGKO cells suggests global chromatin remodeling upon loss of RARγ beyond its direct physical chromatin interactions. The relationship between DNA methylation and histone methylation status at CpG islands is particularly complex, but tri-methylation at H3K27/H3K4 and CpG DNA methylation are generally mutually exclusive (72, 73). The expansion of H3K27me3 and H3K4me3 across CpG regions may also reflect aberrant changes in DNA methylation status and/or DNA methyltransferase recruitment. The concomitant loss of H3K4me3 at the RXRA promoter suggests that RARγ may directly transcriptionally regulate other type II nuclear receptors and indirectly affect other type II nuclear receptors signaling through transcriptional regulation of their binding partners. Notably, RXRA mRNA is greatly reduced in the RARGKO compared to PAR cells and RARγ is bound at the RXRA promoter (Fig. 7*C*, Supporting Information Data S1, Data S2).

We also uncovered a strong enrichment for AP-1 binding motifs, including those recognized by JUN and FOS family members, near RARγ binding in PAR cells (Supporting Information, Fig. S4*E*). This proximity of RARγ and AP-1 sites suggests potential cooperative interactions between RARγ and AP-1 in the potential inhibition of oncogenic pathways as RA was shown to be a negative regulator for AP-1 responsive genes (74). This is also supported by our finding that FOSL1 (FRA1), a subunit of the transcription factor AP-1 and a known driver of metastasis in head and neck cancer (25), is upregulated in RARGKO cells and contains RARγ peaks within its regulatory regions in PAR cells. In addition, we identified significant enrichment of NANOG and SOX2 motifs at H3K4me3 sites in RARGKO cells, further supporting a dedifferentiated phenotype in the RARGKO (75, 76, 77). Thus, loss of RARγ may facilitate a shift toward a more cancer stem cell-like state, potentially contributing to increased tumorigenic and invasive potential in OCSCC. Although we were able to identify significant co-occurrence of RARγ localization and AP-1 binding sites and alterations in associated gene expression and histone modifications, specific functional interrogation of these changes would need to be studied in an *in vivo* model to recapitulate the oral cavity tumor microenvironment.

Our findings highlight the complexity of RARγ function in OCSCC and suggest that targeting RARγ-specific pathways could be a promising therapeutic strategy in the context of oral carcinogenesis. Although pan-RAR activation has been explored in clinical settings (78), selective modulation of RARγ, particularly in combination with epigenetic regulators, may offer more precise control over differentiation and antitumor activity.

Although our study provides significant insights into the role of RARγ in OCSCC, several limitations should be noted. First, our experiments were conducted in a human carcinoma-derived cell line. Additional studies on RARγ functions in normal oral epithelial cells would provide a more comprehensive understanding of transcriptional and epigenetic changes specific to epithelial biology. Future studies should aim to incorporate primary keratinocyte models or normal oral epithelial cells to distinguish between tumor-specific alterations and baseline RARγ functions.

Furthermore, while we identified putative RARγ target genes *via* CUT&RUN, the enrichment of RARγ at distal intergenic and enhancer regions (∼20% of RARγ binding sites) suggests that RARγ may promote higher order chromatin interactions that we were unable to comprehensively assess in this research. Functional validation of such distal regulatory elements through chromatin conformation capture or CRISPR-based enhancer disruption would clarify the regulatory relationships between RARγ and its downstream targets.

In conclusion, we provide a comprehensive analysis of RARγ-mediated transcriptional and epigenetic regulation in OCSCC. We identify key target genes and pathways that define the role of RARγ in maintaining epithelial differentiation. Taken together, our data indicate a multifaceted, context-dependent signaling mechanism for RARγ. Our results emphasize the importance of selective RARγ agonism in therapeutic design and additionally provide a foundation for future investigations into nuclear receptor cross talk in squamous cell carcinoma.

## Experimental procedures

### Cell culture

Human SCC-25 oral squamous carcinoma cells (American Type Culture Collection, CRL-1628) were cultured in DMEM/F-12 (Thermo Fisher Scientific, Cat. 11320033) supplemented with 100U/ml Pen Strep and hydrocortisone (400 ng/ml) and 10% fetal calf serum.

### Immunohistochemistry

All animal experiments and protocols were approved by the Institutional Animal Care and Use Committees (IACUC) of the WCMC. C57BL/6 mouse liver and tongue samples were harvested and fixed in 4% paraformaldehyde buffer (pH 7.4) before paraffin embedding. Slides with 5 μm sections were washed with xylene for 5 min twice, 100% ethanol for 2 min twice, 95% ethanol for 2 min twice, 70% ethanol for 2 min twice, and dH_2_O for 2 min twice. Slides were incubated in diluted antigen unmasking solution (Vector antigen unmasking solution citrate-based, pH 6.0 Cat. H-3300) for 4 min at 176 °C inside a pressure cooker. The slides were treated with 3% hydrogen peroxide in methanol for 15 min to quench unwanted peroxidase activity and then blocked with 10% normal goat serum (Vector Laboratories #S-1000) for 1 h at room temperature (RT). Slides were incubated with primary antibody for RARγ1 (rabbit mAb, Cell Signaling #8965T, 1:50) for 1 h RT and then overnight at 4 °C. After incubation with primary antibody, slides were washed and incubated with 1x goat anti-rabbit IgG secondary antibody, poly HRP conjugate (Invitrogen, #B40962) for 1 h. After PBS washing, slides were incubated with 3,3′-diaminobenzidine substrate (Vector Laboratories), counterstained with hematoxylin (Poly Scientific R&D), and imaged with a Nikon TE2000 inverted fluorescence microscope.

### Generation of the RARG gene KO SCC-25 cells

SCC-25 parental cells (2 x 10^4^) were seeded 1 day before cotransfection with a sgRNA (5′ UGGGCAUGUCCAAGGAAGGU-3′, Synthego) targeting hRARG Exon 5 and CRISPR Cas9 CleanCap mRNA using CRISPRMax Lipofectamine (Thermo Fisher Scientific CMAX00001). Single cell colonies were isolated for culture, and we performed an analysis of RARγ KO efficiency by extracting genomic DNA from subcloned lines, performing PCR across guide RNA target regions, and sequencing the PCR products. The Synthego ICE CRISPR analysis tool was used to predict KO efficiency and visualize sequencing results. Subcloned cell lines with frameshift mutations (Supporting Information Fig. S1*A*) were maintained as putative RARG^−/−^ lines (RARGKO), and we confirmed the loss of RARγ by western blotting. One of the RARGKO clones was selected for RNA-Seq and CUT&RUN experiments.

### Western blotting

Parental and RARGKO cell lines were seeded in triplicate and collected the next day in lysis buffer, briefly sonicated, and soluble fraction was boiled with Laemmli sample buffer (Final concentrations 62.5 mM Tris–HCl, pH 8, 5% β-mercaptoethanol, 2% SDS, 10% glycerol, 0.0025% bromophenol blue). A total of 25 μg protein was loaded per well and SDS-PAGE was run using 10% bis-acrylamide gels. Membranes were blocked with 5% nonfat milk, and incubated overnight at 4 °C with primary antibodies to GAPDH and RARγ1. After secondary antibody incubation membranes were developed with enhanced chemiluminescence solution and imaged on a Bio-Rad instrument. Antibodies used were as follows: RARγ1 (rabbit mAb, Cell Signaling #8965T, 1:1000) and GAPDH (rabbit, ABClonal #AC027, 1:5000). Secondary antibodies: anti-Rabbit IgG (Jackson, 711–135–052, 1:10,000 in 5% milk).

### Genome-wide RNA-sequencing

SCC-25 cells (3 x 10^5^) were seeded in 6-well plates (n = 3 per group). Parental and RARGKO cells were each treated in triplicate with either vehicle (dimethyl sulfoxide, 0.01% final concentration), 1 μM *all-trans* RA, or 1 μM CD1530 for 6 h or 48 h (12 total groups in triplicate). Total RNA was collected from the cells with 350 μl lysis buffer RLT from RNeasy Plus Mini Kit (Qiagen, Cat. No. 74134) with 1:100 addition of β-mercaptoethanol, followed by gDNA removal and extraction according to the kit protocol. Total RNA was submitted to the WCM Genomics Core for Quality Control. Samples with RNA integrity number ≥ 9 were selected and cDNA libraries were prepared with NEB Ultra II Directional RNA Library Prep (plus Poly A isolation module) according to the manufacturer’s instructions. Libraries were paired-end sequenced (2 x 100 cycles) on a NovaSeq X Plus instrument, and raw sequencing reads were processed through Illumina bcl2fastq v.2.20 for demultiplexing and FASTQ conversion. Fastq files were merged per sample and further processed with low-quality (<Q30) base trimming and adapter removal using Cutadapt v.3.5. Reads were pseudoaligned with kallisto using the Homo_sapiens.GRCh38.cdna.all fasta file from Ensembl to generate an index for quantifying transcript abundances. Principle component analysis was performed on kallisto HF5 files outputs using sleuth, and tabular output files were converted with tximport to gene abundance counts for DESeq2 normalization and differential expression analysis. The counts matrix from tximport was prefiltered to exclude low abundance genes, keeping genes that had at least 10 counts in at least three samples. For analysis of genes with altered expression in RARGKO *versus* PAR in the absence of added agonist, we combined + vehicle samples from the 6 h experiment and the 48 h experiment into 1 group for each genotype (n = 6 per group). Differentially expressed genes were identified using DESeq2. Cutoffs were set to p-adjusted < 0.05 and |log_2_(Fold Change)| > 0.3 unless otherwise specified. Promoter-site analysis for differentially expressed genes was performed using HOMER (47) (Hypergeometric Optimization of Motif EnRichment) v5.1 findMotif.pl. Volcano plots were generated with EnhancedVolcano. GSEA was performed with clusterProfiler (31) on gene expression ranked by DESeq2 stat and plotted using profileplyr and ggplot2. ORA was performed using clusterProfiler with the full set of genes expressed in SCC-25 used as background.

DESeq2 pairwise results were compared to identify sets of genes with different transcriptional responses to ligand in an RARG^+/+^ *versus* null background and sets of genes that were differentially expressed in an RARG null background, but also unresponsive to RA or CD1530 treatment in an RARG^+/+^ background.

### CUT&RUN

We performed CUT&RUN (79) using the CUTANA ChIC/CUT&RUN Kit (EpiCypher, #14–1048) and corresponding protocol (v.4.1) with some modifications. PAR and RARGKO SCC-25 cells were seeded in triplicate in 150 cm dishes and lightly fixed the next day for 2 min with 0.1% methanol-free formaldehyde directly in the dish before quenching with 20x Glycine for 2 min. Cells were trypsinized and collected, washed with 1% fetal calf serum in 1XPBS, and resuspended in Nuclei Extraction Buffer (EpiCypher) for 10 min on ice. Nuclear extraction efficiency was assessed before centrifuging samples 300*g* for 5 min at 4 °C and resuspending at 4 million nuclei/ml. 400,000 nuclei per sample were added to activated ConA beads and processed according to the EpiCypher manual, with the addition of detergents recommended by EpiCypher’s CUTANA Cross-linking Protocol (v1.9, 2022). SNAP-CUTANA K-MetStat Panel of d-nucleosomes was spiked into IgG, H3K4me3, and H3K27me3 samples for on-target assessment and experimental quality control. The spike-in alignment was performed using shell script written by Dr Bryan Venters and downloaded from the EpiCypher website. ConA bead-bound nuclei were incubated with antibodies to either IgG (Rabbit, EpiCypher #13–0042, 1:50), H3K4me3 (Rabbit, EpiCypher #13–0041, 1:50), H3K27me3 (Thermo Fisher Scientific, #MA5-11198, 1:10), or RARγ1 (Rabbit, Cell Signaling #8965T, 1:50). *Escherichia coli* DNA from the kit was spiked in with the MNase reaction stop buffer for normalization. Before DNA elution, samples were decrosslinked overnight with SDS and proteinase K. Yield concentrations were checked with a Qubit fluorometer using 1x-High Sensitivity dsDNA reagent (Thermo Fisher Scientific Cat. No. Q33230). Library preparation was performed with the IDT-xGen kit and samples were sequenced using a NextSeq 2000 with Paired End 50 bp reads. Samples were prepared in triplicate, and one replicate for RARγ binding in PAR was excluded from downstream analysis due to sample prep issues and poor-quality reads.

Sequencing reads were trimmed with Trim Galore! (80) and quality-checked with FastQC. Reads were aligned to hg38 with Bowtie2 (81), with parameters adjusted to allow dovetailing. Sorted BAM files were filtered to remove mitochondrial alignments, sequences with MAPQ scores <30, and fragments >120 bp with SAMtools and further processed with picard (82) MarkDuplicates to mark and remove PCR duplicates. Separate alignments to *E*. *coli* M-12 were performed to calculate single-scaler normalization factors for each sample based on the fraction of uniquely mapped *E*. *coli* reads. Deeptools bamCoverage was used to generate spike-in normalized bigWig files for IGV visualization. For all track windows, replicates were overlaid and autoscaled by antibody target, with RARγ and IgG samples grouped together for scaling. Paired end, uniquely aligned, filtered BAM files for technical replicates were merged, and narrow peak calling was performed on BAM files for samples targeting RARγ using MACS3 (42) in paired end mode with the parameters --nomodel, --shiftsize −100, --extsize 200, --keep-dup all. Sites called with -log_10_(q) > 5 were included in the peak set, and IgG alignments were used as negative controls. The parental RARγ peak set was filtered beyond MACS3 called peaks with q > 5 to remove peaks that overlapped (using bedtools intersect with overlap threshold set to 0.1) peaks called with RARGKO samples targeting RARγ.

The R package ChIPseeker (43) was used to plot Feature Distributions and Genomic Coverage (Fig. 5, Supporting Information Fig. S3). For differential enrichment analysis of histone PTMs, MACS3 was used as above on unmerged processed BAM files to produce narrowPeak files for H3K4me3 and broadPeak files for H3K27me3. DiffBind in DESeq2 mode was performed for each of H3K4me3 (narrowPeak) and H3K27me3 (broadPeak) peak sets in RARGKO *versus* PAR in R, using IgG BAM files as negative controls for grey list generation and *minOverlap = 2*. Fold enrichment (FE) was calculated as the ratio of log2 (normalized read concentration) values between RARGKO and PAR. Regions with FE > 0 and FDR < 0.05 were considered enriched in RARGKO, and regions with FE < 0 and FDR < 0.05 were considered depleted in RARGKO with respect to PAR cells.

### Motif analysis

*De novo* motif analysis was performed using HOMER and MEME suite (45) command line tools, using the hg38 genome and promoter resources downloaded directly from HOMER. Known HOMER motif analysis was performed using findMotifGenome.pl. Position frequency matrices (PFMs) from the JASPAR 2024 non-redundant vertebrates subcollection and the nonredundant set of HOCOMOCO v12 motifs (83) (derived from the CORE set) were downloaded in meme and homer (*p* value=0.0005) formats. These PFMs and log odds scoring were used for all motif analysis unless otherwise specified.

Background files for 300 bp and 500 bp lengths were generated for MEME Suite tasks using fasta-get-markov with randomly selected gene intervals with similar GC content to target CUT&RUN peak sets, first-order modeling. Background BED files for HOMER tasks were similarly generated using the “homer2 background” program with the full peak sets for RARγ, H3K4me3, and H3K27me3 as references for GC%.

For motif analysis of RARγ peaks categorized by corresponding gene response to RARGKO or RARγ agonism, the MEME suite program AME (46) was used to compare each subset of RARγ peaks with all other RARγ CUT&RUN binding sites (-log_10_q.val >5) using Fisher’s test for motif enrichment and average log odds for sequence scoring. AME identification of motifs in differentially H3K4me3-enriched and H3K27me3-enriched regions was similarly performed (with Rank Sum test for enrichment), using regions with FE > 1 compared with FE < 0 and FE < −1 compared to FE > 0 (FDR < 0.05) regions to capture more extreme shifts in H3 lysine modifications in RARGKO samples (Supporting Information Fig. S4*D*).

CentriMo (84) from MEME suite was ran in local mode to identify motif colocalization at RARγ summit-centered 500 bp regions (Supporting Information Fig. S4, *E* and *F*). The HOMER program findMotifs.pl was used to identify motif enrichment within the human promoter regions of subsets of differentially expressed genes in response to agonist treatment (Supporting Information Fig. S5*C*).

### Repeat analysis

The HOCOMOCO RARG.H13CORE.1.PSM.A motif was used as a template for gapped motif enrichment analysis. The motif PFM was trimmed to the 6-mer 5′-RGKTCR-3′ region and direct (DR), everted (ER), and inverted (IR) repeats were generated with N spacers ranging from 0 to 12. HOMER known motif enrichment with ZOOPS scoring (zero or one occurrence per sequence) coupled with binomial calculations was performed using these custom PFMs for peaks associated with genes differentially expressed in response to RARγ agonism and/or loss, with -log_10_ (*p* value) used to compare relative enrichment of different repeats within the RARγ peak subsets. The R package universalmotif was also used to identify and score putative sequence matches to repeats within the peak sets. A cutoff threshold of 0.7 ∗ maximum log odds score was used to identify sequence hits.

For significantly enriched spacings between RARγ RARE primary motif sites and secondary motifs within the HOCOMOCO and JASPAR nonredundant datasets, the MEME suite program SpaMo (61) was ran with settings -minscore −0.75 -margin 100 -pseudo 0.0001 -shared 1 -overlap 6 -cutoff 0.00001 for each set of peaks grouped by repeat type and expression changes in RARGKO *versus* PAR. RARγ primary motifs were identified using the spaced repeats generated along with PWMs obtained from the top RARγ-binding MinTerm sequence set (MinSeq) from Bhimsaria, *et al*. (Supporting Information Data S5) (59).

### Statistical analysis

Statistical analysis was performed in R and GraphPad Prism. RNA-seq differential expression was performed using DESeq2 in R using Wald tests for each gene with Benjamini–Hochberg corrections to adjust for false discovery during multiple comparison analysis. Significant values are identified as p-adjusted values with significant differences defined as less than an alpha of 0.05 unless a stricter cutoff is specified. MACS3 peak calling uses local Poisson modeling to generate *p* values, with q values calculated using Benjamini–Hochberg procedure. All analyses in R were performed using R Statistical Software version 4.4.1 (R Core Team 2024). All other packages and tools were installed with Anaconda and implemented using Python ≥ 3.8 and in-house shell scripts. The coding scripts are available upon request.

## Data availability

RNA-Sequencing data is available from GEO under accession #GSE295225. CUT&RUN data is available from GEO under accession #GSE295226.

## Supporting information

This article contains supporting information (59).

## Conflict of interest

The authors declare that they have no conflicts of interest with the contents of this article.
