# Supplementary material for: Multiple modes of transcriptional regulation by the nuclear hormone receptor RARγ in human squamous cell carcinoma
Source: J Biol Chem. 2025 Nov 20;302(1):110965. doi: 10.1016/j.jbc.2025.110965 (PMC12774775; doi:10.1016/j.jbc.2025.110965)
Supplement: Supporting Information Data 7 [file mmc7.pdf]

Supporting Information for

Multiple modes of transcriptional regulation by the nuclear hormone receptor RARγ in human squamous cell carcinoma

Helen Hoxie[1,3], Xiao-Han Tang[1,2], and Lorraine Gudas[1,2,3]*

Department of Pharmacology, Weill Cornell Medicine, New York, NY, USA [1], Sandra and Edward Meyer Cancer Center, Weill Cornell Medicine, New York, NY, USA [2], and Weill Cornell Graduate School of Medical Sciences, Cornell University, New York, NY, USA [3]

* Corresponding Author: Lorraine Gudas

Email: *ljgudas@med.cornell.edu*

This file includes:

- Figures S1 to S5 and Legends
- Tables S1 to S3
- Legends for Datasets S1 to S6

Other supporting materials for this manuscript include the following:

SI Datasets S1 to S6

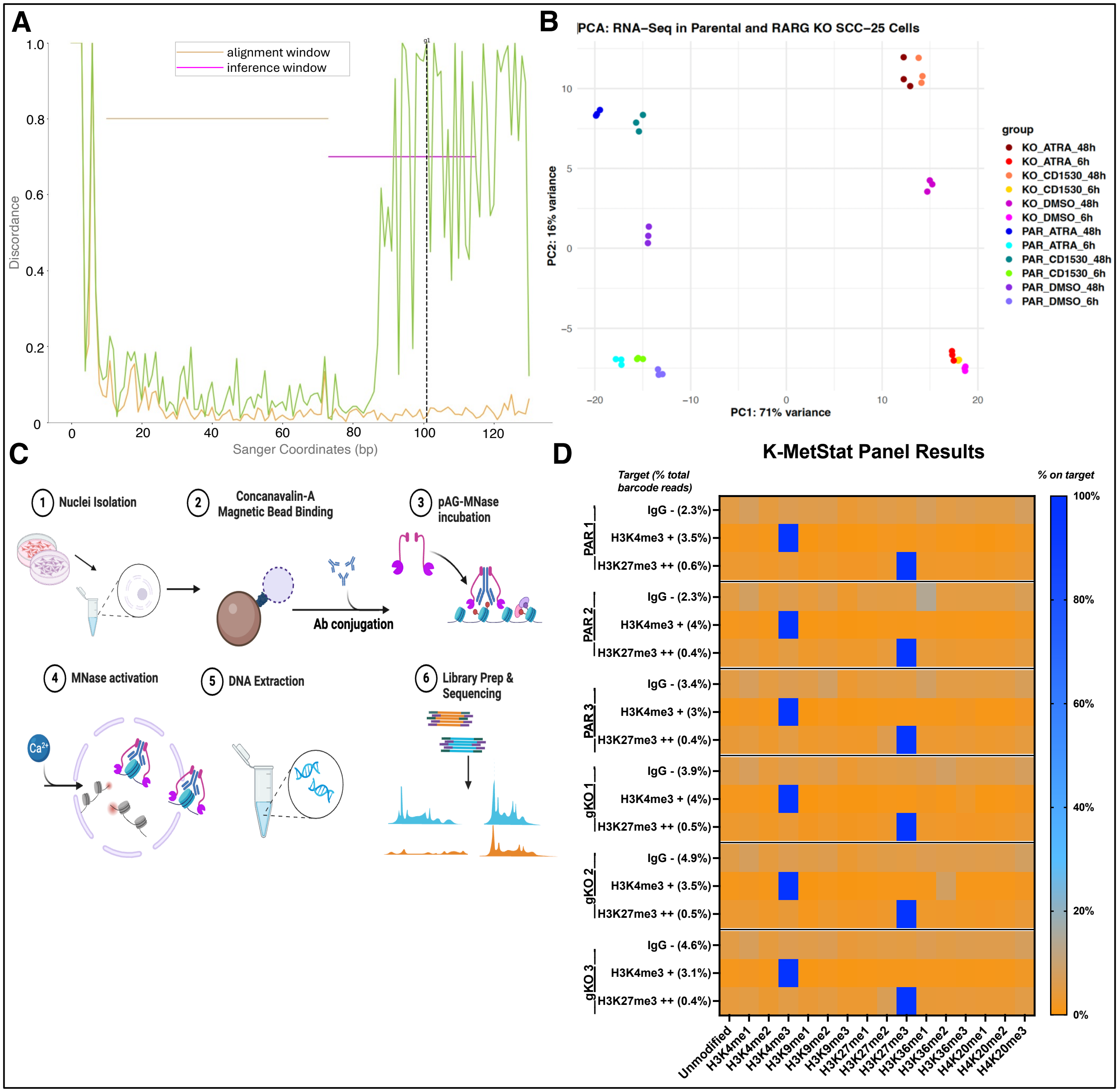

Fig. S1. Methods to identify genomic targets of RARγ signaling**. A,** Synthego ICE confirmation of indel mutation in SCC-25 RARGKO-3-2 cells; **B**, RNA-Seq sample to sample distances at 6h and 48h hour time points, n=3 per condition, with KO = RARGKO SCC-25 line, PAR = Parental SCC-25 line, and treatments at 1 μM concentrations; **C,** Overview of SCC-25 Parental and RARGKO cell sample processing for CUT&RUN; **D,** K-MetStat Panel analysis of spike-in control reads confirming histone modification specificity of H3K4me3, H3K27me3, and IgG (negative control) antibodies in the experimental conditions used.

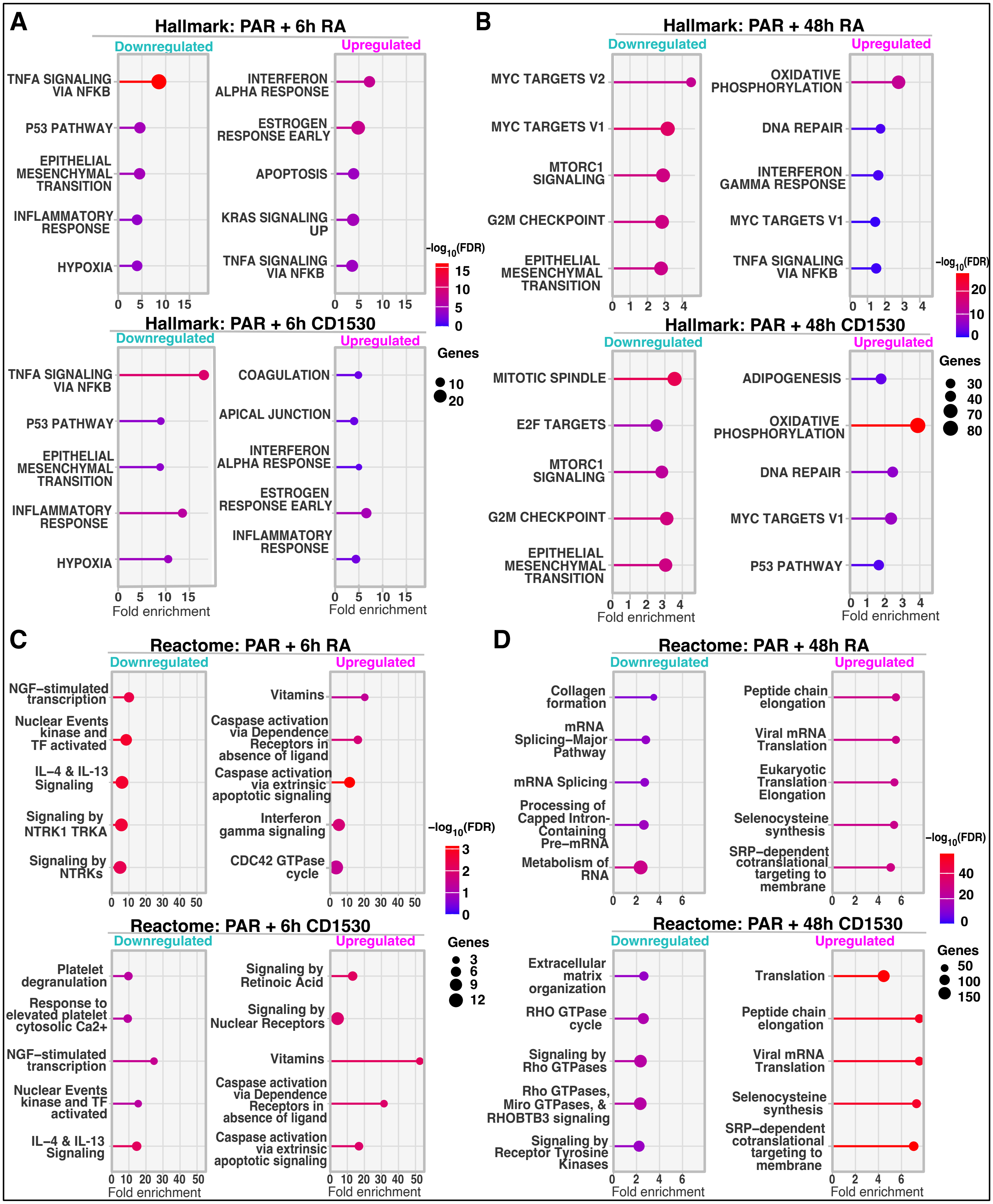

Fig. S2. Overrepresentation pathway analysis (ORA) for genes differentially expressed in response to RARγ agonist treatment. **A-B**, ORA using the Hallmark (MSigDB 6.1) database of genes differentially expressed (p.adj < 0.01, $|log_2FC| > 0.3$) in response to agonist treatments in PAR at 6h (**A**) and 48h (**B**); **C-D,** ORA with the Reactome (v.64) database of genes differentially expressed (p.adj < 0.01, $|log_2FC| > 0.3$) in response to agonist treatments in PAR at 6h (**C**) and 48h (**D**). Graphs within panels are scaled together, and the set of genes expressed in SCC-25 cells from RNA-Seq analysis was used as background.

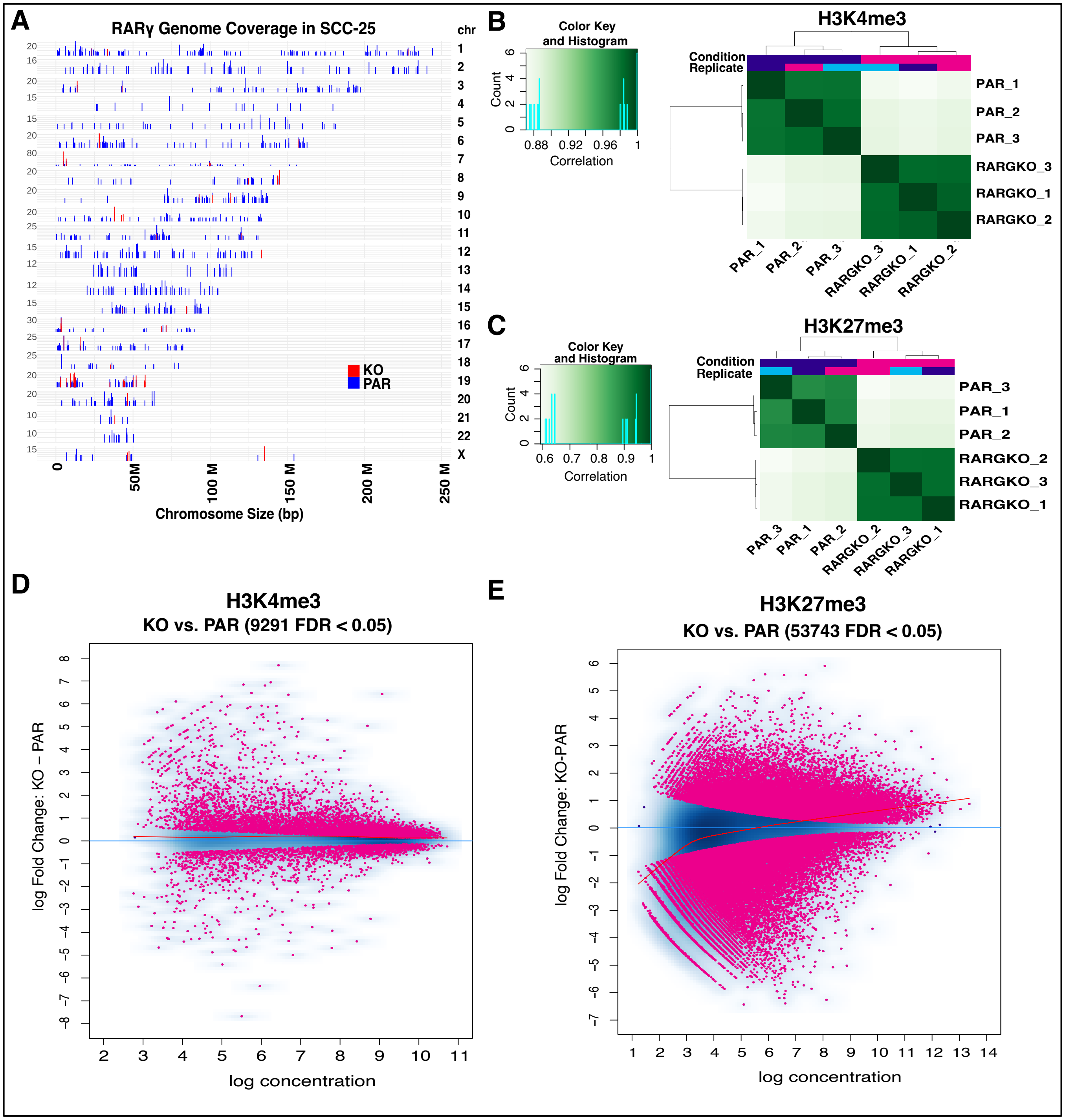

Fig. S3. CUT&RUN in SCC-25 cells. **A,** Genome wide coverage of peaks called with MACS3 for RARγ. Chromosomes are arranged alphanumerically from top to bottom, with autoscaling for maximum signal at each chromosome; **B-C,** Correlation heatmaps showing sample-sample Pearson correlations computed from normalized read counts across peaks and clustered by correlation distance for **B,** H3K4me3 and **C,** H3K27me3 peaksets; **D-E,** MA plots showing results of DiffBind analysis for H3K4me3 (**D**) and H3K27me3 (**E**) binding in SCC-25 cells using normalization factors generated from E. coli spike-in reads and the DESeq2-based negative binomial generalized linear model (GLM) to identify differentially bound sites. Differentially bound sites between RARGKO and PAR with an FDR < 0.05 are plotted as pink dots, with positive log_2FoldChange (FE) values indicating sites that have increased binding in RARGKO over PAR.

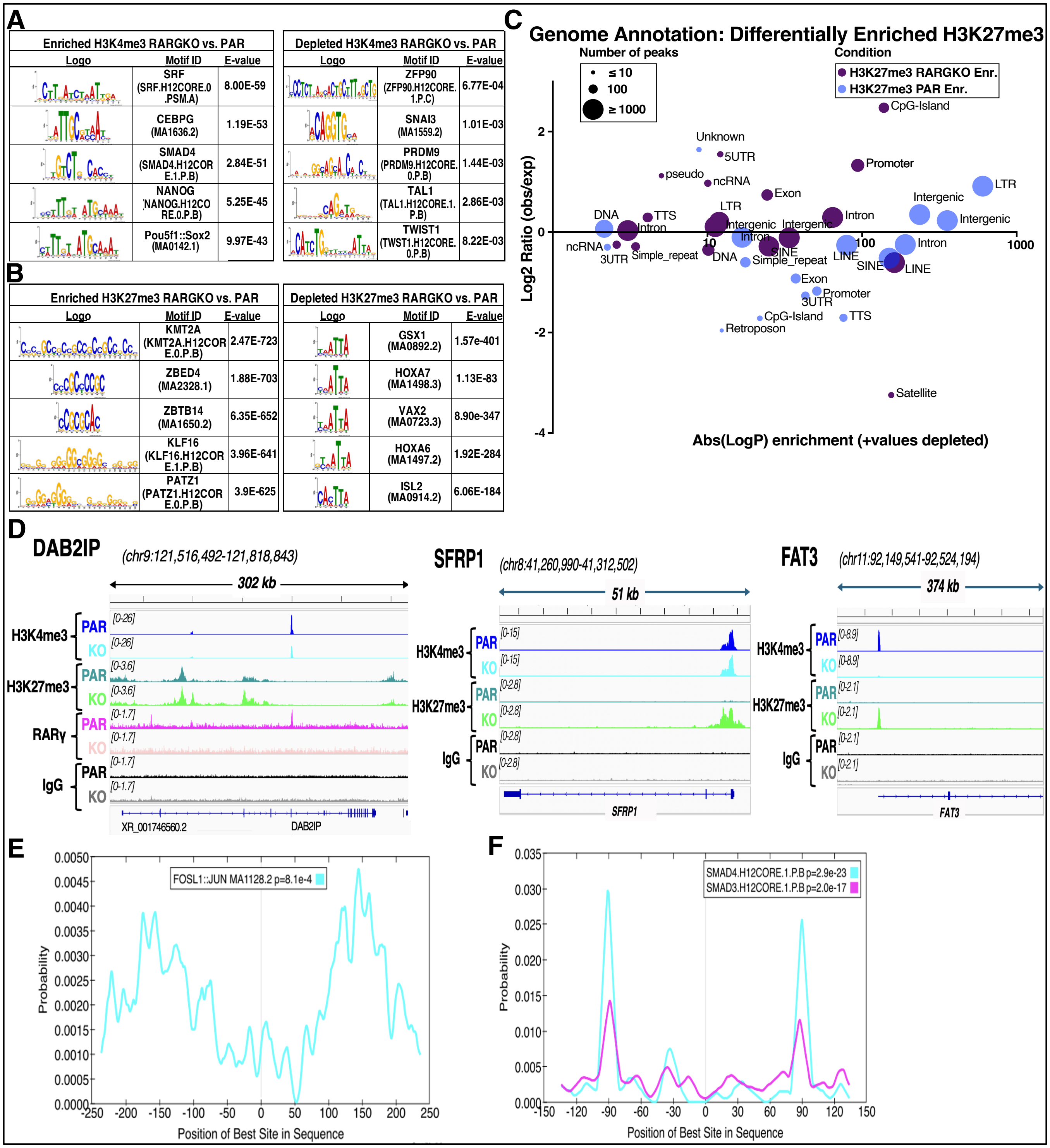

Fig. S4. Differential binding profiles for H3K4me3, H3K27me3, and RARγ in SCC-25 RARGKO vs. PAR. **A**, Differential motif enrichment analysis using MEME-suite tool AME of H3K4me3 sites, comparing sites with H3K4me3 enriched (FE >1) in RARGKO vs. PAR (FE < -1); **B,** AME analysis of H3K27me3 differentially bound sites. Sequences were scored using average log odds and enrichment determined using ranksum based on FE; **C**, Genome Ontology Enrichment Analysis for H3K27me3 peaks enriched (FDR < 0.05, FE >1) or depleted (FDR < 0.05, FE < -1) in RARGKO compared to PAR; **D,** Tracks displaying examples of differential histone modification enrichment in RARGKO vs. PAR, with an example of non-overlapping occupancy between H3K4me3 and H3K27me3 but opposite shifts in signal (*left*), an example of bivalency established in RARGKO *(middle*), and an example of monovalent switching (*right*); **E**, Representative AP-1 JASPAR motif co-localization with RARγ binding sites. Top 1000 peaks were centered on peak summit and extended 250bp in both directions before running Centrimo analysis in local mode; **F,** Smad3/4 motif co-localization with RARγ binding sites containing and centered on a 5’RGKTCR3’ RARγ half-site motif.

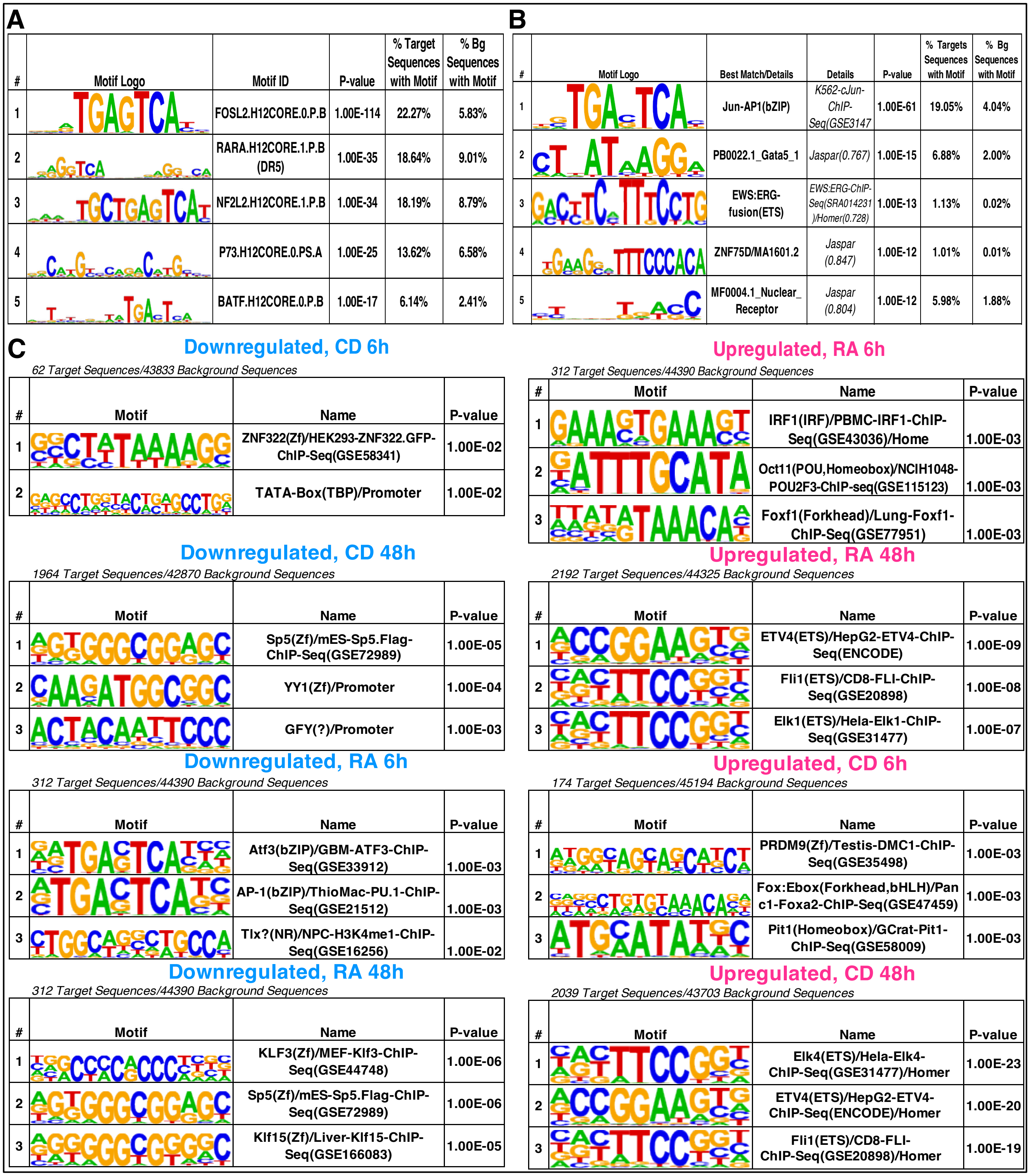

A

#	Motif Logo	Motif ID	P-value	% Target Sequences with Motif	% Bg Sequences with Motif
1		FOSL2.H12CORE.0.P.B	1.00E-114	22.27%	5.83%
2		RARA.H12CORE.1.P.B (DR5)	1.00E-35	18.64%	9.01%
3		NF2L2.H12CORE.1.P.B	1.00E-34	18.19%	8.79%
4		P73.H12CORE.0.PS.A	1.00E-25	13.62%	6.58%
5		BATF.H12CORE.0.P.B	1.00E-17	6.14%	2.41%

B

#	Motif Logo	Best Match/Details	Details	P-value	% Targets Sequences with Motif	% Bg Sequences with Motif
1		Jun-AP1(bZIP)	*K562-cJun-ChIP-Seq(GSE3147*	1.00E-61	19.05%	4.04%
2		PB0022.1_Gata5_1	*Jaspar(0.767)*	1.00E-15	6.88%	2.00%
3		EWS:ERG-fusion(ETS)	*EWS:ERG-ChIP-Seq(SRA014231)/Homer(0.728)*	1.00E-13	1.13%	0.02%
4		ZNF75D/MA1601.2	*Jaspar (0.847)*	1.00E-12	1.01%	0.01%
5		MF0004.1_Nuclear_Receptor	*Jaspar (0.804)*	1.00E-12	5.98%	1.88%

C

Downregulated, CD 6h

62 Target Sequences/43833 Background Sequences

#	Motif	Name	P-value
1		ZNF322(Zf)/HEK293-ZNF322.GFP-ChIP-Seq(GSE58341)	1.00E-02
2		TATA-Box(TBP)/Promoter	1.00E-02

Upregulated, RA 6h

312 Target Sequences/44390 Background Sequences

#	Motif	Name	P-value
1		IRF1(IRF)/PBMC-IRF1-ChIP-Seq(GSE43036)/Home	1.00E-03
2		Oct11(POU,Homeobox)/NCIH1048-POU2F3-ChIP-seq(GSE115123)	1.00E-03
3		Foxf1(Forkhead)/Lung-Foxf1-ChIP-Seq(GSE77951)	1.00E-03

Downregulated, CD 48h

1964 Target Sequences/42870 Background Sequences

#	Motif	Name	P-value
1		Sp5(Zf)/mES-Sp5.Flag-ChIP-Seq(GSE72989)	1.00E-05
2		YY1(Zf)/Promoter	1.00E-04
3		GFY(?)/Promoter	1.00E-03

Upregulated, RA 48h

2192 Target Sequences/44325 Background Sequences

#	Motif	Name	P-value
1		ETV4(ETS)/HepG2-ETV4-ChIP-Seq(ENCODE)	1.00E-09
2		Fli1(ETS)/CD8-FLI-ChIP-Seq(GSE20898)	1.00E-08
3		Elk1(ETS)/Hela-Elk1-ChIP-Seq(GSE31477)	1.00E-07

Downregulated, RA 6h

312 Target Sequences/44390 Background Sequences

#	Motif	Name	P-value
1		Atf3(bZIP)/GBM-ATF3-ChIP-Seq(GSE33912)	1.00E-03
2		AP-1(bZIP)/ThioMac-PU.1-ChIP-Seq(GSE21512)	1.00E-03
3		Tlx?(NR)/NPC-H3K4me1-ChIP-Seq(GSE16256)	1.00E-02

Upregulated, CD 6h

174 Target Sequences/45194 Background Sequences

#	Motif	Name	P-value
1		PRDM9(Zf)/Testis-DMC1-ChIP-Seq(GSE35498)	1.00E-03
2		Fox:Ebox(Forkhead,bHLH)/Panc1-Foxa2-ChIP-Seq(GSE47459)	1.00E-03
3		Pit1(Homeobox)/GCrat-Pit1-ChIP-Seq(GSE58009)	1.00E-03

Downregulated, RA 48h

312 Target Sequences/44390 Background Sequences

#	Motif	Name	P-value
1		KLF3(Zf)/MEF-Klf3-ChIP-Seq(GSE44748)	1.00E-06
2		Sp5(Zf)/mES-Sp5.Flag-ChIP-Seq(GSE72989)	1.00E-06
3		Klf15(Zf)/Liver-Klf15-ChIP-Seq(GSE166083)	1.00E-05

Upregulated, CD 48h

2039 Target Sequences/43703 Background Sequences

#	Motif	Name	P-value
1		Elk4(ETS)/Hela-Elk4-ChIP-Seq(GSE31477)/Homer	1.00E-23
2		ETV4(ETS)/HepG2-ETV4-ChIP-Seq(ENCODE)/Homer	1.00E-20
3		Fli1(ETS)/CD8-FLI-ChIP-Seq(GSE20898)/Homer	1.00E-19

Fig. S5. HOMER motif analysis of RARγ binding sites in SCC-25 PAR. **A**, Known and **B,** de novo analysis of top 2,000 RARγ binding peaks by MACS3 integer score; **C,** HOMER motif analysis of human promoter regions for genes differentially expressed ($|Log_2(FoldChange)| > 0.3$ & p.adj < 0.01) in response to agonist treatment.

Table S1: Expression of RAR and RXR isotypes in SCC-25 cell lines. Length-normalized counts from DESeq2 are shown for each isotype, with standard deviation in parentheses. All values are rounded to the nearest integer.

	**Time:**	**6h**			**48h**		
	**Drug:**	**Vehicle**	**1μM CD1530**	**1μM RA**	**Vehicle**	**1μM CD1530**	**1μM RA**
**SCC-25 Parental**	**RARA**	778 (±15)	732 (±15)	616 (±21)	716 (±35)	593 (±32)	576 (±13)
	**RARB**	50 (±20)	127 (±14)	186 (±5)	92 (±8)	153 (±28)	341 (±36)
	**RARG**	3481 (±73)	3281 (±56)	3369 (±123)	3387 (±109)	2396 (±73)	2148 (±82)
	**RXRA**	22657 (±426)	21010 (±126)	21083 (±403)	23288 (±1062)	16594 (±333)	15172 (±701)
	**RXRB**	1052 (±217)	1143 (±53)	1125 (±157)	789 (±556)	776 (±457)	1020 (±85)
**SCC-25 RARGKO**	**RARA**	937 (±43)	922 (±28)	817 (±27)	846 (±14)	636 (±24)	598 (±25)
	**RARB**	22 (±2)	60 (±4)	71 (±8)	106 (±14)	168 (±6)	220 (±4)
	**RARG**	327 (±29)	341 (±31)	341 (±13)	351 (±12)	325 (±36)	304 (±31)
	**RXRA**	14272 (±131)	13545 (±76)	13438 (±70)	14626 (±397)	10284 (±492)	9991 (±551)
	**RXRB**	992 (±183)	1108 (±117)	1039 (±41)	986 (±275)	873 (±76)	884 (±57)

Table S2: Software Packages and Versions Used in this Study

**Software**	**Installation Source**	**Version**
kallisto	homebrew	0.51.1
cutadapt	anaconda3	3.5
EnhancedVolcano	R (BioConductor)	1.24.0
DESeq2	R (BioConductor)	1.46.0
eulerr	R (CRAN)	7.0.2
TxDb.Hsapiens.UCSC.hg38.knownGene	R (BioConductor)	3.20.0
tximport	R (BioConductor)	1.34.0
msigdbr	R (BioConductor)	7.5.1
GenomicRanges	R (BioConductor)	1.58.0
GenomicFeatures	R (BioConductor)	1.58.0
GenomeInfoDb	R (BioConductor)	1.42.3
dplyr	R (CRAN)	1.1.4
ComplexHeatmap	R (BioConductor)	2.22.0
clusterProfiler	R (BioConductor)	4.14.6
ChIPseeker	R (BioConductor)	1.42.1
AnnotationDbi	R (BioConductor)	1.68.0
purrr	R (CRAN)	1.0.4
reactome.db	R (BioConductor)	1.89.0
universalmotif	R (BioConductor)	1.24.2
tidyr	R (CRAN)	1.3.1
deeptools	anaconda3	3.5.5
deeptoolsintervals	anaconda3	0.1.9
bowtie2	anaconda3	2.5.4
bedtools	homebrew	2.31.1
Trim Galore!	anaconda3	0.6.10
samtools	anaconda3	1.21

macs3	anaconda3	3.0.2
bedops	homebrew	2.4.41
python	local	≥ 3.8
Homer	UCSD site	5.1
hg38	UCSD site	7
bedtools	homebrew	2.31.1
meme-suite	anaconda3	5.5.5
visNetwork	R (CRAN)	2.1.2
samtools	anaconda3	1.21
picard	.jar from Broad Institute git repository	3.3.0
R	CRAN Repository	≥4.0
Bioconductor	BiocManager	≥3.0
Biostrings	R (BioConductor)	2.74.1
biomaRt	R (BioConductor)	2.62.1
fgsea	R (BioConductor)	3.21
Diffbind	R (BioConductor)	3.16.0

Table S3. Gene Symbols Annotated with Full Names and Corresponding Protein Names

Gene Symbol	Full Gene Name	Protein Symbol	Protein Name
**RARA**	Retinoic acid receptor alpha	RARα	Retinoic acid receptor alpha
**RARB**	Retinoic acid receptor beta	RARβ	Retinoic acid receptor beta
**RARG**	Retinoic acid receptor gamma	RARγ	Retinoic acid receptor gamma
**RXRA**	Retinoid X receptor alpha	RXRα	Retinoid X receptor alpha
**SPRR1B**	Small proline rich protein 1B	SPRR1B	Small proline-rich protein 1B
**MAGEA3**	Melanoma antigen family A3	MAGE-A3	Melanoma-associated antigen 3
**MAGEC2**	Melanoma antigen family C2	MAGE-C2	Melanoma-associated antigen C2
**KRT4**	Keratin 4	K4	Keratin, type II cytoskeletal 4
**IRX1**	Iroquois homeobox 1	IRX1	Iroquois-class homeodomain protein [illegible]
**MUC4**	Mucin 4, cell surface associated	MUC4	Mucin-4
**SMAD3**	SMAD family member 3	SMAD3	Mothers against decapentaplegic [illegible]
**SMAD6**	SMAD family member 6	SMAD6	Mothers against decapentaplegic [illegible]
**SHH**	Sonic hedgehog signaling molecule	SHH	Sonic hedgehog protein
**NOTCH1**	Notch receptor 1	Notch1	Neurogenic locus notch homolog protein [illegible]
**NOTCH3**	Notch receptor 3	Notch3	Neurogenic locus notch homolog protein [illegible]
**JAG2**	Jagged canonical Notch ligand 2	Jag2	Protein jagged-2
**DLL1**	Delta like canonical Notch ligand 1	DLL1	Delta-like protein 1
**JAG1**	Jagged canonical Notch ligand 1	Jag1	Protein jagged-1
**KRT6A**	Keratin 6A	K6A	Keratin, type II cytoskeletal 6A
**KRT14**	Keratin 14	K14	Keratin, type I cytoskeletal 14
**KRT7**	Keratin 7	K7	Keratin, type II cytoskeletal 7
**KRT80**	Keratin 80	K80	Keratin, type II cytoskeletal 80
**ITGB1**	Integrin subunit beta 1	ITGβ1	Integrin beta-1
**ITGA6**	Integrin subunit alpha 6	ITGα6	Integrin alpha-6
**ITGB3**	Integrin subunit beta 3	ITGβ3	Integrin beta-3

**ITGB6**	Integrin subunit beta 6	ITGβ6	Integrin beta-6
**HOXA1**	Homeobox A1	HOXA1	Homeobox protein Hox-A1
**HOXA3**	Homeobox A3	HOXA3	Homeobox protein Hox-A3
**CYP26A1**	Cytochrome P450 family 26 subfamily A	CYP26A1	Cytochrome P450 26A1
**DHRS3**	Dehydrogenase/reductase 3	DHRS3	Short-chain dehydrogenase/reductase 3
**JUNB**	JunB proto-oncogene, AP-1	JunB	Transcription factor Jun-B
**NDRG1**	N-myc downstream regulated gene 1	NDRG1	Protein NDRG1
**ITGA1**	Integrin subunit alpha 1	ITGα1	Integrin alpha-1
**PLAU**	Plasminogen activator, urokinase	uPA	Urokinase-type plasminogen activator
**WNT10A**	Wnt family member 10A	Wnt10a	Protein Wnt-10a
**KRT78**	Keratin 78	K78	Keratin, type II cytoskeletal 78
**KRT86**	Keratin 86	K86	Keratin, type II cytoskeletal 86
**NRL**	Neural retina leucine zipper	NRL	Neural retina-specific leucine zipper
**SLC2A6**	Solute carrier family 2 member 6	GLUT6	Glucose transporter type 6
**SLCO3A1**	Solute carrier organic anion transporter	OATP3A1	Organic anion transporting polypeptide
**SUSD1**	Sushi domain containing 1	SUSD1	Sushi domain-containing protein 1
**TAPBP**	TAP binding protein	Tapasin	TAP-binding protein
**DUSP6**	Dual specificity phosphatase 6	DUSP6	Dual specificity protein phosphatase 6
**EGR2**	Early growth response 2	Egr-2	Early growth response protein 2
**RICTOR**	RPTOR independent companion of	RICTOR	Rapamycin-insensitive companion of
**POU2F2**	POU class 2 homeobox 2	Oct-2	POU domain, class 2, transcription factor
**PTGES**	Prostaglandin E synthase	PTGES	Prostaglandin E synthase
**CYP26B1**	Cytochrome P450 family 26 subfamily B	CYP26B1	Cytochrome P450 26B1
**FOXF1**	Forkhead box F1	FoxF1	Forkhead box protein F1
**FOXF2**	Forkhead box F2	FoxF2	Forkhead box protein F2
**DUSP9**	Dual specificity phosphatase 9	DUSP9	Dual specificity protein phosphatase 9
**DAB2IP**	DAB2 interacting protein	DAB2IP	DAB2-interacting protein
**TP63**	Tumor protein p63	p63	Tumor protein p63
**SMAD4**	SMAD family member 4	SMAD4	Mothers against decapentaplegic homolog 4
**CCNA1**	Cyclin A1	CCNA1	Cyclin-A1
**THBS1**	Thrombospondin 1	TSP1	Thrombospondin-1
**EGR1**	Early growth response 1	Egr-1	Early growth response protein 1
**KRT5**	Keratin 5	K5	Keratin, type II cytoskeletal 5
**LAMB3**	Laminin subunit beta 3	LAMB3	Laminin subunit beta-3
**CNR1**	Cannabinoid receptor 1	CNR1	Cannabinoid receptor 1
**CEL**	Carboxyl ester lipase	CEL	Bile salt-dependent lipase
**CYSRT1**	Cysteine-rich tail protein 1	CYSRT1	Cysteine-rich tail protein 1
**IRF1**	Interferon regulatory factor 1	IRF1	Interferon regulatory factor 1
**GPR37L1**	G protein-coupled receptor 37 like 1	GPR37L1	G-protein coupled receptor 37-like 1
**PPARG**	Peroxisome proliferator-activated	PPARγ	Peroxisome proliferator-activated
**NAV2**	Neuron navigator 2	NAV2	Neuron navigator 2
**RBP1**	Retinol binding protein 1	RBP1	Cellular retinol-binding protein I
**NR2F1**	Nuclear receptor subfamily 2 group F	COUP-TFI	Chicken ovalbumin upstream promoter
**AMIGO2**	Adhesion molecule with Ig-like domain	AMIGO2	Amphoterin-induced gene and ORF 2
**STK17B**	Serine/threonine kinase 17b	STK17B	Serine/threonine-protein kinase 17B
**FOS**	Fos proto-oncogene, AP-1 transcription	c-Fos	Proto-oncogene protein c-Fos

**JUN**	Jun proto-oncogene, AP-1 transcription [illegible]	c-Jun	Transcription factor AP-1
**ZEB1**	Zinc finger E-box binding homeobox 1	ZEB1	Zinc finger E-box-binding homeobox 1
**SNAI2**	Snail family transcriptional repressor 2	Slug	Protein Slug
**STAT1**	Signal transducer and activator of [illegible]	STAT1	Signal transducer and activator of [illegible]
**STAT5**	Signal transducer and activator of [illegible]	STAT5	Signal transducer and activator of [illegible]
**NANOG**	Nanog homeobox	NANOG	Homeobox protein NANOG
**SOX2**	SRY-box transcription factor 2	SOX2	Transcription factor SOX-2
**POU5F1**	POU class 5 homeobox 1	Oct4	Octamer-binding transcription factor 4
**FOXA1**	Forkhead box A1	FoxA1	Forkhead box protein A1
**ELF3**	E74 like ETS transcription factor 3	ELF3	ETS-related transcription factor Elf-3
**CTNND1**	Catenin delta 1	p120- [illegible]	Catenin delta-1
**ITGA3**	Integrin subunit alpha 3	ITGα3	Integrin alpha-3
**SERPINE1**	Serpin family E member 1	PAI-1	Plasminogen activator inhibitor 1
**MMP7**	Matrix metallopeptidase 7	MMP-7	Matrix metalloproteinase-7
**MMP13**	Matrix metallopeptidase 13	MMP-13	Matrix metalloproteinase-13
**MMP14**	Matrix metallopeptidase 14	MMP-14	Matrix metalloproteinase-14
**COL7A1**	Collagen type VII alpha 1 chain	COL7A1	Collagen alpha-1(VII) chain
**COL12A2**	Collagen type XII alpha 2 chain	COL12A2	Collagen alpha-2(XII) chain
**COL4A2**	Collagen type IV alpha 2 chain	COL4A2	Collagen alpha-2(IV) chain

Description of Additional Supporting Information Files
File name: SI Dataset S1 (.xlsx)
Description: DESeq2 Results for RNA-Seq in SCC-25 cells.

File name: SI Dataset S2 (.xlsx)
Description: RARγ peaks called using MACS3 from merged replicates 2 & 3 in narrow peak mode, after exclusion of RARGKO RARγ peaks. For all peaks, $-\log_{10}(q) > 5$.

File name: SI Dataset S3 (.xlsx)
Description: Differentially bound peaks for H3K4me3 (Sheet 1) and H3K27me3 (Sheet 2) between SCC-25 PAR and RARGKO ($|\log_2(FC)| > 1$, FDR < 0.05).

File name: SI Dataset S4 (.xlsx)
Description: RARγ binding sites at super enhancer regions identified in SCC-25 cells (downloaded from SEdb) and associated target genes. Corresponding mean expression, $\log_2(FC)$, and p.adj for agonist treatments vs. PAR + DMSO and RARGKO vs. PAR is listed for each target gene, and the nearest associated gene to the peak summit is also listed. $\text{Log}_2(FC)$ values highlighted in blue or red reflect significant expression changes (p.adj < 0.05).

File name: SI Dataset S5 (.txt)
Description: Position frequency matrices (MEME) for RARγ motif analysis, including those identified in Bhimsaria, D. et. al., *Nat Commun* 2023.

File name: SI Dataset S6 (.xlsx)
Description: DESeq2 results for SCC-25 cells categorized by response to agonist treatment and annotated with RARγ peak information (non-overlapping, no RARγ binding in RARGKO. The RARγ peak closest to the TSS is reported for genes with RARγ interactions at their regions. Sheet labeled "info" provides detailed descriptions for each of the Sheets, and gene lists expanding on Table 1 in the main manuscript are included at the end. Gene Transcription Responses are classified by DE direction in RARGKO vs. PAR, DE direction in PAR after Agonist treatment in PAR, DE direction in RARGKO after agonist treatment in RARGKO.
